# Photoredox Catalysis Using Heterogenized Iridium Complexes**

**DOI:** 10.1002/chem.202101651

**Published:** 2021-07-22

**Authors:** Kelly L. Materna, Leif Hammarström

**Affiliations:** ^1^ Department of Chemistry-Ångström Laboratories Uppsala University Box 523, SE 75120 Uppsala Sweden

**Keywords:** heterogenized iridium, photocatalysis, photoredox catalysis, sustainable chemistry

## Abstract

Heterogenized photoredox catalysts provide a path for sustainable chemical synthesis using highly tunable, reusable constructs. Here, heterogenized iridium complexes as photoredox catalysts were assembled via covalent attachment to metal oxide surfaces (ITO, ZrO_2_, Al_2_O_3_) in thin film or nanopowder constructs. The goal was to understand which materials provided the most promising constructs for catalysis. To do this, reductive dehalogenation of bromoacetophenone to acetophenone was studied as a test reaction for system optimization. All catalyst constructs produced acetophenone with high conversions and yields with the fastest reactions complete in fifteen minutes using Al_2_O_3_ supports. The nanopowder catalysts resulted in faster and more efficient catalysis, while the thin film catalysts were more robust and easily reused. Importantly, the thin film constructs show promise for future photoelectrochemical and electrochemical photoredox setups. Finally, all catalysts were reusable 2–3 times, performing at least 1000 turnovers (Al_2_O_3_), demonstrating that heterogenized catalysts are a sustainable catalyst alternative.

## Graphical abstract

**Heterogenized iridium catalysts** were synthesized to generate reusable, robust photoredox catalysts. The catalyst architecture, in nanopowder or thin film forms, was studied to see how it affected the catalytic performance on three different metal oxide surfaces. Reductive dehalogenation of bromoacetophenone to acetophenone was studied as the model photoredox reaction, and all catalysts showed successful product generation. Al_2_O_3_‐based catalysts were found to be the most robust and reusable catalysts in both architectures, with at least ∼1000 turnovers and reusable three times.

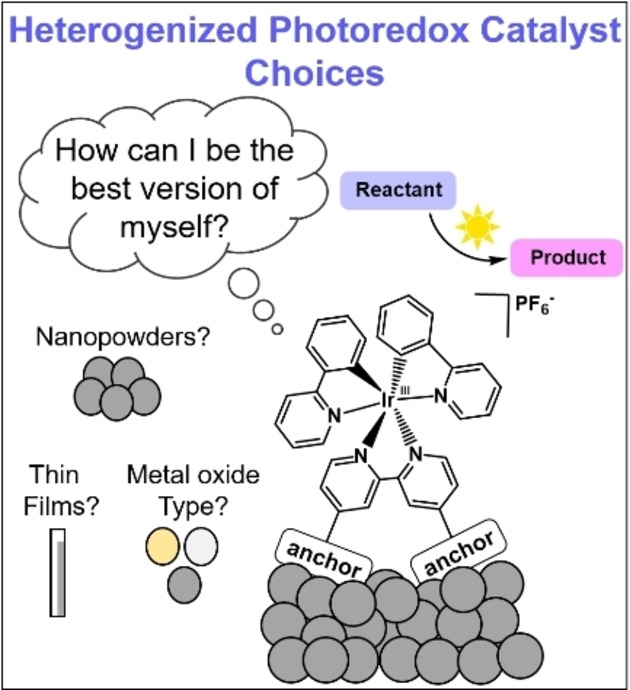

## Introduction

Due to the active occurrence of climate change and its impending consequences, the world today *requires* renewable energy sources to drive components of daily life.^[1]^ Much progress has been made in the solar fuels and photovoltaics communities, where sunlight is used to generate electricity using solar cells, and produce renewable fuels, such as H_2_, using photoelectrochemical cells.^[2]^ For example, water‐splitting dye sensitized photoelectrochemical cells (WS‐DSPEC) incorporate molecular photosensitizers to harvest sunlight and perform charge separation and use molecular catalysts to drive fuel production; these molecular components are covalently attached to semiconducting metal oxide surfaces. The molecular nature of the components in WS‐DSPECs makes them highly tunable, while the metal oxide surface provides a robust platform for photoelectrocatalysis. However, because the devices are complex, with multiple molecular components and a semiconducting surface that participates during catalysis, fuel production can be challenging as multiple charges must be accumulated for the catalyst to turnover.^[3]^

**Scheme 1:**
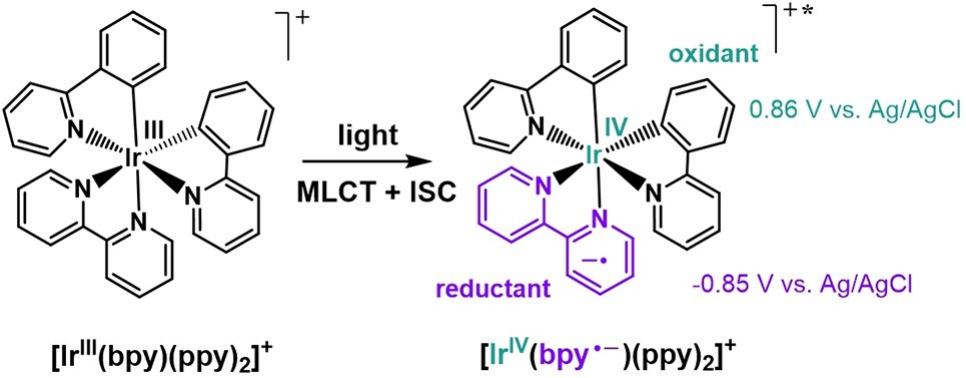
Example reaction of photoredox catalyst [Ir^III^(bpy)(ppy)_2_]^+^ with light, where ppy=2‐phenylpyridine, and bpy=2,2′‐bipyridine.^[6]^ The catalyst undergoes a metal to ligand charge transfer (MLCT) and intersystem crossing (ISC) to form the charge separated excited state species. Excited state potentials displayed in the scheme are based on literature data collected in acetonitrile under argon.^[6]^

Complementary to this, the photoredox catalysis field has demonstrated that a variety of chemicals (natural products, pharmaceuticals, feedstock chemicals, etc.) can be produced in an environmentally friendly fashion under mild conditions using common household light bulbs.^[4]^ Upon illumination, molecular photoredox catalysts generate long‐lived excited states (ns‐μs), which are able to react with organic substrates in a reaction mixture. Typically in photoredox catalysis, catalysts are homogenous, molecular species such as ruthenium, iridium, or copper coordination complexes; these inorganic coordination complexes undergo a metal to ligand charge transfer (MLCT) and intersystem crossing (ISC) upon excitation, producing long‐lived excited states, which are simultaneously highly reducing and oxidizing in nature (Scheme 1).[^4e^, ^4f^, ^5^] Given this duality of the excited state, the complexes can act as effective photoredox catalysts in a library of organic transformations.

Furthermore, homogeneous molecular photoredox catalysts have the advantage of being highly tunable due to their molecular nature, allowing a variety of organic transformations to be studied. However, as they are usually in the same phase as the organic substrate and product, a purification step is needed to separate them from the reaction mixture. To make the catalyst easier to separate from the reaction mixture, heterogeneous solid‐state materials have also been used as photoredox catalysts.^[4a]^ These catalysts are both robust and easily reusable catalysts; although, they are less tunable than molecular, homogeneous catalysts.

**Figure 1:**
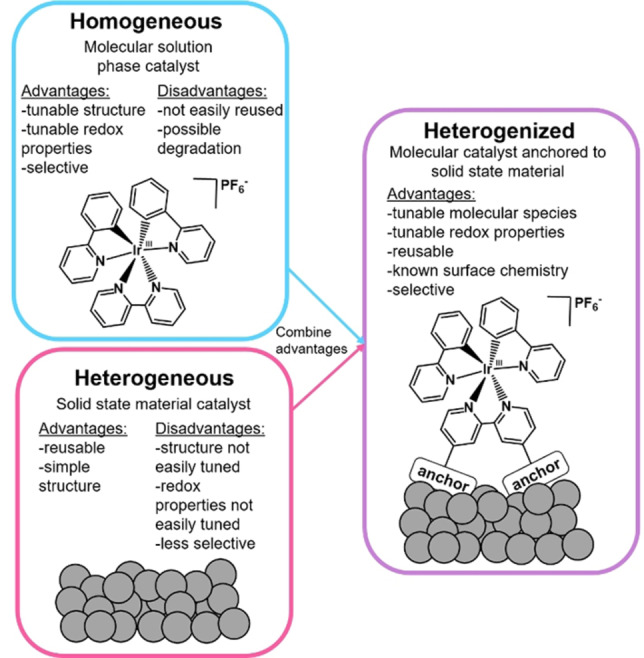
Differences between homogeneous, heterogeneous, and heterogenized catalysts.

To assemble a catalyst that has the advantages of both a homogeneous and heterogeneous one, inspiration can be taken from the solar fuels field, by designing a photoredox catalyst that is molecular in nature, but covalently bound to a solid‐state support, called a *heterogenized* catalyst (Figure 1).[^2e^, ^2g^, ^7^] To covalently attach the molecular catalyst to the solid‐state support, a surface anchor is used, which is incorporated into the molecular catalyst structure. Upon immobilization, this reacts with a metal oxide (MOx) solid‐state support to form a covalent bond; this method is well‐established in the solar fuels field and dye sensitized solar cell (DSSC) literature.^[8]^ By assembling a catalyst in this fashion, we gain the tunability of a molecular catalyst and also the reusability of heterogeneous catalyst to design the ultimate environmentally‐friendly photoredox catalyst.

In the photoredox catalysis field, this idea has not been extensively explored. A few examples exist using derivatives of tris(2,2'‐bipyridine)‐ruthenium coordination complexes immobilized onto silica,^[9]^ glass wool,^[10]^ or polymer^[11]^ supports. Other unique examples have also been tested such as Rose Bengal^[12]^ dye or perylene diimide (PDI)^[13]^ molecules immobilized on silica, and porphyrins immobilized onto cotton threads.^[14]^ Heterogenized iridium photoredox catalysts are even less explored, with only a few examples existing where polypyridyl‐based iridium complexes have been incorporated into polymer supports;^[15]^ note that in polymer supports, iridium leaching can be an issue leading to catalyst instability.^[15b]^ All of these studies demonstrate a promising future for heterogenized photoredox catalysts since they provide tunable and reusable catalysts; however, there lacks an organized set of guidelines for how to design a heterogenized photoredox catalyst.

To address this issue, we will perform a systematic study to analyze the effect of the catalyst support in both composition and architecture. To do this, polypyridyl iridium coordination complexes will be used and immobilized onto three different metal oxide supports in the form of nanopowders or thin films. The results here will provide a strong foundation to the heterogenized photoredox catalysis field by identifying what materials are optimal when using these catalysts. The overall goal is to develop a heterogenized catalyst that is highly functional, easily reusable, tunable, and useful in a variety of photoredox applications. In this study, photoredox catalysis for the reductive dehalogenation of 2‐bromoacetophenone (BrAPN) to acetophenone (APN) was chosen as the model reaction to analyze the effect of the photoredox catalyst's support, in both content (composition of the metal oxide) and in architecture (thin film or nanopowder). This reaction is advantageous as it is simple, well‐known, and is easy to follow using ^1^H NMR spectroscopy, making it an ideal reaction for initial catalytic studies.^[16]^ Furthermore, the product APN is non‐toxic and highly applicable in the perfume industry. Ultimately, we aim to generate an initial understanding of what materials and assembly methods provide the most functional and robust heterogenized photoredox catalysts by performing a baseline, systematic study. Once this foundation is established, additional reaction and substrate scopes can be analyzed in future work to test for increased applicability.

## Results and Discussion

### Catalyst design and rationale

**Figure 2:**
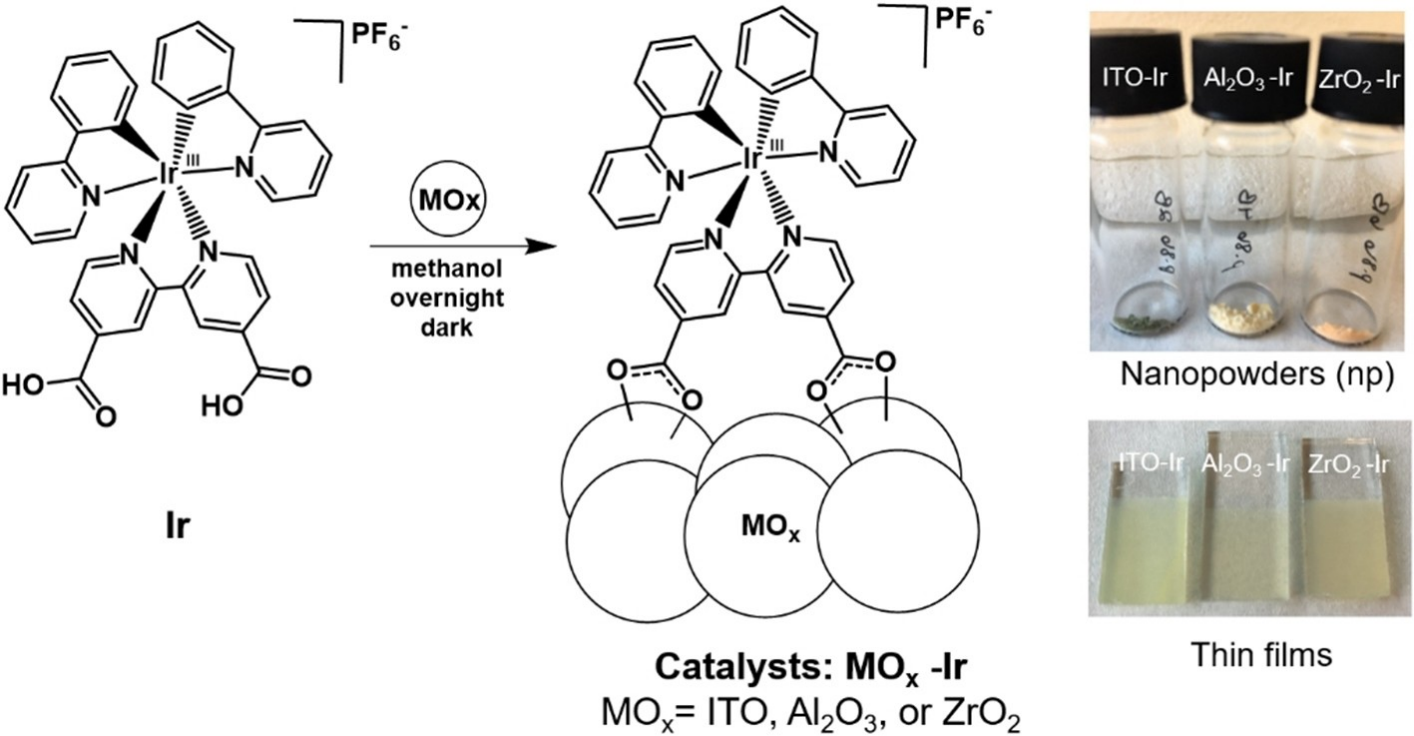
Structure of **Ir** (left complex) and proposed structure of heterogenized **Ir** (MO_x_‐**Ir**). Preparation method for catalysts is depicted. The metal oxide nanopowders or thin films are soaked in an **Ir**/methanol solution overnight in the dark to provide the heterogenized catalysts (MOx‐**Ir**). Pictures of the nanopowder catalysts are shown in the top right and thin films in the bottom right.

To design the iridium catalyst, we were inspired by the varieties of tris(2‐phenylpyridine)iridium and bis(2‐phenylpyridine)(2,2’‐bipyridine)iridium coordination complexes that are widely used in homogeneous photoredox catalysis.[^4c^, ^4d^, ^4e^, ^4f^, ^6^, ^17^] To be able to bind the complex to a metal oxide surface, carboxylic acid surface anchors were incorporated in the catalyst structure (**Ir**, Figure 2) by replacing a 2,2′‐bipyridine ligand of the latter complex for a 2,2′‐bipyridine‐4,4′‐dicarboxylic acid ligand. Carboxylic acids are commonly used surface anchors in the solar fuels and dye sensitized solar cell (DSSC) communities, as they can form covalent surface bonds to metal oxide surfaces.^[8]^ In addition, the photophysical properties of **Ir** has been previously studied.^[18]^ Absorption features in the visible region and a long excited state lifetime (69.5 ns in acetonitrile) make it a good candidate for photoredox catalysis.

### Ir synthesis and characterization

To prepare the heterogenized catalysts, **Ir** was synthesized via a previously reported procedure.^[18]^ Briefly, **Ir** was synthesized via reaction of [Ir(ppy)_2_(μ‐Cl)]_2_ with 2,2′‐bipyridine‐4,4′‐dicarboxylic acid in dichloromethane followed by workup with ammonium hexafluorophosphate, NH_4_PF_6_ (more details in the Supporting Information). **Ir** was characterized by proton nuclear magnetic resonance (^1^H NMR), UV‐Vis, photoluminescence, and attenuated total reflectance Fourier transform infrared (ATR‐FTIR) spectroscopic measurements supporting that **Ir** was successfully synthesized (see Supporting Information). The UV‐Vis spectrum of **Ir** shows a MLCT band at 370 nm and a broad band at 497 nm (Figure S10). ATR‐FTIR confirms the presence of the carboxylic acid anchoring group, having a signature peak at 1709 cm^−1^, suggesting the presence of a C=O group (Figure S19). The photoluminescence spectrum after excitation at 370 nm shows a band with maximum at 635 nm, typical for this class of complexes (Figure S36).[^18^, ^19^]

### Metal oxide rationale

Three different metal oxide (MOx) supports were chosen to be examined: Al_2_O_3_, ZrO_2_, and ITO. Al_2_O_3_ and ZrO_2_ are wide band gap semiconductors, while ITO is a conductive metal oxide. Since Al_2_O_3_ (8.45–9.9 eV) and ZrO_2_ (5 eV) have wide band gaps and high energy conduction bands,^[20]^ and **Ir**'s redox potentials lie far from the valence and conduction band potentials, **Ir** should not be able to inject charges into the metal oxide upon excitation. As a result, no redox events should occur between the catalyst and metal oxide. Therefore, the metal oxide should only act as a support for the molecular catalyst and should not actively participate in catalysis (unlike semiconductors with lower energy conduction bands, such as TiO_2_ and ZnO where charge injection could occur). This allows us to probe **Ir**'s activity in photoredox catalysis with less interference from the surface. To increase catalyst applicability in future heterogenized setups, ITO (indium doped tin oxide) was also chosen as it is a conductive metal oxide, having a continuous electronic band structure‐ (i. e. no band gap); ITO itself should not undergo light induced reactions, and thus, should mainly act as a catalyst support (although electron injection from the catalyst might occur[^2h^, ^21^]). Further, ITO is advantageous as it can be applied as an electrode material in (photo)electrochemical setups in future studies,^[7b]^ which would allow sacrificial reagents to be eliminated from reactions as charges could be provided by the counter electrode in an external circuit; this would create a more environmentally‐friendly way to do photoredox catalysis.

### Metal oxide architectures

The effect of the metal oxide architecture was also examined, either as nanopowders or thin films. Some differences in reactivity, reusability, and applicability may be found with the different architectures (Table 1). For example, nanopowders can be stirred in a reaction, minimizing concentration gradients by enabling fast catalyst transport to the BrAPN substrate, which should result in quick reactions. In contrast, thin films reactions have no catalyst diffusion or transport via stirring as the catalyst is immobilized onto a metal oxide thin film annealed to a glass slide. The substrate must be transported to the catalyst within the film via stirring and diffusion; thus, slower reactions with thin films are expected. The quantity (equivalents) of **Ir** in the reactions with nanopowder supports can be more easily modified than thin films, as more nanopowder MO_x_‐**Ir** can simply be added to the reaction. Whereas, to increase the quantity (equivalents) of catalyst with the thin films (if the catalyst loading is already maximized), a larger geometric surface area and/or thicker films would be needed to add more MOx‐**Ir**. Since the films are placed in the reaction vessel, the vessel must be large enough to fit a larger film, which could require a custom designed vessel. Regarding reusability, thin films are advantageous since they can be easily removed from the reaction without losing any catalyst. In contrast, nanopowder‐based catalysts require centrifugation or filtration to remove the catalyst and several rinsing steps, which could result in some catalyst loss over time, thus limiting the long‐term reusability. Thin films can be easier to characterize by spectroscopic methods (UV‐Vis, ATR‐FTIR, TCSPC, XPS) as they are transparent in nature, possibly having a more evenly distributed catalyst loading, and can be studied as prepared unlike nanopowders. Finally, thin films have the more applicable architecture when it comes to future electrochemical or photoelectrochemical setups as an FTO‐coated glass electrode is used to support the thin film in these devices and provides a conductive contact in the electrical circuit.

**Table 1:** Advantages and disadvantages of thin films versus nanopowders.

Thin Films		Nanopowders	
Advantages:	Disadvantages:	Advantages:	Disadvantages:
Easily removed with tweezers from reaction	Slower reactions‐ no diffusion or convective transport (via stirring) of catalyst	Faster reactions‐catalyst diffusion and convective transport via stirring	Sample loss due to centrifugation and rinsing
			
No sample loss	Less catalyst loading (due to size restriction of film)	Potential for more catalyst loading	Harder to characterize catalyst
			
Easy to characterize catalyst	Extra preparation step	One step preparation	
			
Applicable in (photo) electrochemical setups

### Preparation of heterogenized nanopowder catalysts

To prepare the heterogenized catalysts, approximately 25 mg of nanopowder (Al_2_O_3_, ZrO_2_, ITO) was stirred in the presence of 2.5 mL of a 0.25 mM solution of **Ir** in methanol overnight in the dark. Post sensitization, the catalyst was centrifuged off from the sensitization solution and rinsed three times with methanol via subsequent rinsing and centrifugation steps. Finally, the catalyst was dried under vacuum for several hours, affording the MOx‐**Ir** nanopowders (Figure 2).

### Preparation of heterogenized thin film catalysts

To prepare the thin film based catalysts, metal oxide thin films were first prepared. One layer of metal oxide paste was doctor bladed onto FTO‐coated glass slides, followed by annealing at high temperatures for several hours (see Supporting Information for further details). The thin films were then soaked in a 0.1 mM **Ir** sensitization solution in methanol overnight, rinsed with methanol three times, and air‐dried (Figure 2).

### Catalyst characterization

**Figure 3:**
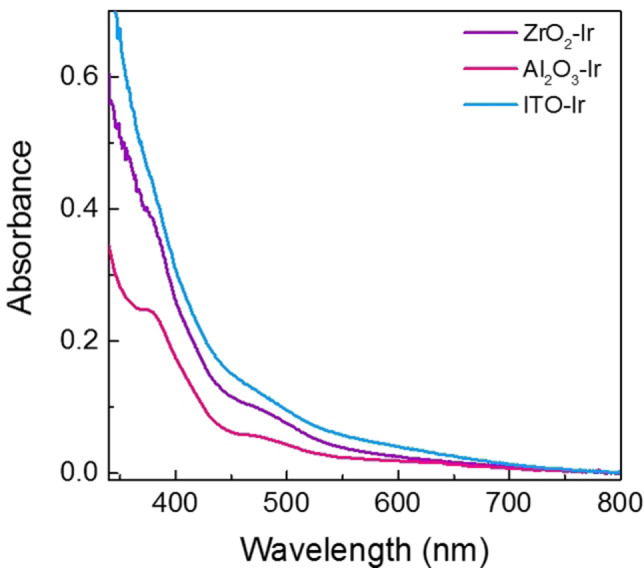
UV‐Vis spectra of thin films of ZrO_2_‐**Ir** (purple), Al_2_O_3_‐**Ir** (pink), ITO‐**Ir** (blue).

Several spectroscopic methods were used to characterize the catalysts. First, UV‐Vis spectroscopy was used to analyze the **Ir** surface loadings on the nanopowders and thin films. For the nanopowders, the depletion method was used to quantify the **Ir** surface loadings, which is a well‐established procedure in the solar fuels field.[^7b^, ^22^] To do this, the UV‐Vis spectrum of the sensitization solution was collected before and after exposure to the nanopowders to get the initial and final iridium concentrations in the solutions using the molar extinction coefficient, ϵ(370 nm)=8730 cm^−1^ M^−1^ (Figure S10). After sensitization of the nanopowders and centrifugation, a decrease in absorbance at 370 nm was observed in the supernatant, suggesting that the iridium complex in solution had bound to the nanopowders (Figure S12). The difference in iridium concentration before and after sensitization was calculated and approximated to be the loading on the surface. For the samples, an average loading of 6.0±0.9 nmol/mg for Al_2_O_3_‐**Ir**, 7.1±0.8 nmol/mg for ZrO_2_‐**Ir**, and 6.7±0.9 nmol/mg for ITO‐**Ir** was obtained (Table S1). For the thin films, UV‐Vis spectra was collected directly on the thin films, showing similar absorption features to the **Ir** in solution, suggesting the molecular structure had been retained upon surface binding (Figure 3). The loadings (Γ) were obtained using the formula, Γ(mol cm^−2^ )=A(λ)/ (1000ϵ), where A is the absorbance at wavelength λ, and ϵ is the molar extinction coefficient at wavelength λ.^[23]^ Average loadings were calculated and found to be 28.4±6.9 nmol/cm^2^ for Al_2_O_3_‐**Ir**, 31.1±8.7 nmol/cm^2^ for ZrO_2_‐**Ir**, and 35.8±7.0 nmol/cm^2^ for ITO‐**Ir** (Table S2). Finally, if larger catalyst loadings were desired, they could be potentially increased by increasing the thin film thickness, increasing the concentration of the catalyst sensitization solution, or by changing the sensitization solution solvent.

To further characterize the heterogenized catalysts, ATR‐FTIR, and XPS were collected on the nanopowders and thin films. In the ATR‐FTIR spectra, C=C and C=N aromatic stretches at 1607 cm^−1^ and 1583 cm^−1^, respectively, are observed in both **Ir** powder and MO_x_‐**Ir** samples, suggesting that the molecular structure is retained upon binding (Figure S20–21). In addition, the C=O stretch in **Ir** at 1709 cm^−1^ disappears upon surface binding, suggesting that the carboxylic acid anchor binds in a bridging bidentate binding mode to covalently attach the catalyst the metal oxide supports.^[8]^ X‐ray photoelectron spectroscopic (XPS) measurements also show characteristic peaks for **Ir** in the iridium 4 f region at 61.8 eV and 64.7 eV, which match well with the **Ir** powder XPS spectrum, supporting the presence of iridium on the metal oxides (Figure S26–27). Furthermore, to demonstrate that the carboxylic acid group in **Ir** was necessary for covalent attachment to the metal oxide support, we tested to see if an iridium complex without a carboxylic acid surface anchor would also attach to the metal oxide support. To do this, Ir(ppy)_3_ (Tris[2‐phenylpyridinato‐C2,N]iridium(III), which has no surface anchor, was soaked in the presence of an Al_2_O_3_ thin film in methanol. Post soaking, no iridium absorption feature were detected in the UV‐Vis spectrum of the thin film (Figure S11B), suggesting that the carboxylic acid anchor was necessary to covalently attach **Ir** to the supports. Based on the UV‐Vis, ATR‐FTIR, and XPS spectroscopic data, **Ir** has successfully bound to the metal oxides and is likely a molecular complex on the surface.

### Electrochemistry

To ensure that **Ir** is capable of reducing the BrAPN substrate, cyclic voltammetric (CV) measurements were performed on ITO‐**Ir** to get the reduction potentials of the heterogenized catalyst. The CVs in acetonitrile showed quasi‐reversible reduction and oxidation waves, at −1.93 V vs. Fc/Fc^+^ and 0.98 V vs. Fc/Fc^+^, respectively (Figure S32), while the CV of BrAPN showed an irreversible reduction at −1.73 V vs. Fc/Fc^+^(Figure S33). These potentials confirm that the reduced **Ir^−^** should be thermodynamically able to reduce BrAPN.

### Excited state potentials

Excited state potentials for **Ir** were estimated from the UV‐Vis absorption spectrum, photoluminescence spectrum, and ground state redox potentials using Weller approximations.^[24]^ Intersection of the normalized absorption and emission spectra give an estimated transition energy from the ground state to the lowest excited state (E_0‐0_) of 2.21 eV (Figure S36). Using the Weller approximations, excited state potentials were estimated to be −1.23 V vs. Fc/Fc^+^ for Ir^+^/Ir* (E^0^(Ir^+^/Ir*)=E^0^(Ir^+^/Ir)−E_0‐0_) and 0.28 V vs. Fc/Fc^+^ for Ir*/Ir^−^ (E^0^(Ir*/Ir^‐^ )=E^0^(Ir/Ir^−^)+E_0‐0_). Thus, the **Ir** excited state is thermodynamically unable to reduce the substrate during photoredox catalysis. Furthermore, one should note that triethanolamine (TEOA) is also present during the reductive dehalogenation reactions as a sacrificial electron donor and proton source. The TEOA reduction potential is estimated to be 0.3 V vs. Fc/Fc^+^ [0.7 V vs. SCE, Ref. [25]] but as its' oxidation is irreversible, this value should be taken as an approximation and not a fixed value; hence, the **Ir** excited state is most likely quenched by TEOA during photoredox catalysis.

### Time correlated single photon counting

Prior to catalytic testing, the catalyst excited state lifetimes were quantified using time correlated single photon counting (TCSPC) measurements to ensure they did not change significantly upon surface binding and had long enough lifetimes for photoredox reactions (>1 ns needed). To verify this, thin film catalysts were used for these measurements and were placed in the sample holder as a dry thin film in air. Nanopowders were not tested, as the particles settle to the bottom of a cuvette without stirring making catalyst excitation challenging. For the measurements, **Ir** was excited with a 470 nm laser pulse, and the photoluminescence from the excited state was monitored over time via collection of single photon events (Figure S34).^[26]^ Decays were fit to mono or biexponential functions and the results can be found in Table S5. Using monoexponential fits, τ_1_=220 ns was found for ZrO_2_‐**Ir**. This lifetime is similar to that of the homogeneous version of **Ir** in acetonitrile, having τ_1_=210 ns. Biexponential fits were used for Al_2_O_3_‐**Ir** and ITO‐**Ir** as these did not fit well to single exponential decays; this could be due to surface inhomogeneity or non‐innocent surface behavior. For these surfaces, an average excited state lifetime (τ_average_) of 1.5 ns was found for ITO‐**Ir**, and 3.3 ns for Al_2_O_3_‐**Ir**. The overall trend in catalyst excited state lifetime was then ZrO_2_‐**Ir>**Al_2_O_3_‐**Ir>**ITO‐**Ir**. It is possible that the conductivity of ITO interferes some with the **Ir** excited state (via electron injection into ITO), causing a faster excited state decay,[^2h^, ^21^] while potential surface trap states in Al_2_O_3_, may also shorten the lifetimes.^[27]^ We note that the lifetimes reported here should be taken as estimates as the measurements were collected and fitted under shorter time scales than the catalyst lifetime; longer timescales will be measured in future work to obtain more precise values during mechanistic studies. Recall, the main goal was to demonstrate that the catalyst excited state persisted long enough to participate in catalytic reactions. Furthermore, all samples show excited state lifetimes longer than 1 ns, which the minimum lifetime needed to react with a substrate diffusing in a reaction mixture. Thus, all catalysts are good candidates for photoredox tests.

### Catalyst stability

As catalyst desorption can occur in heterogenized systems,^[8]^ a quick check was performed prior to catalytic tests to ensure that **Ir** was stable on the surface in the reaction solvent, acetonitrile. No catalyst loss from the metal oxide was observed after soaking MOx‐**Ir** in acetonitrile overnight (Figure S17). In addition, no light induced desorption was observed when the metal oxide was illuminated with a white light lamp in acetonitrile (Figure S17).

### Initial catalytic tests

**Scheme 2:**
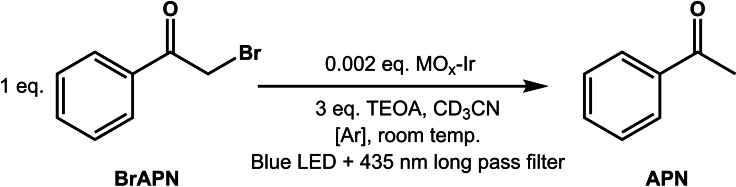
Reaction conditions for reductive dehalogenation of bromoacetophenone (BrAPN) to acetophenone (APN).

Since **Ir** had been successfully characterized on the three surfaces, and had properties promising for photoredox catalysis, initial reductive dehalogenation tests were conducted. A reaction scheme is depicted in Scheme 2 for the reductive dehalogenation of BrAPN to APN. We were inspired by results from a Tris(bipyridine)Ru(II) metal‐organic framework (MOF) photoredox catalyst reported to catalyze reductive dehalogenation reactions.^[16a]^ In the reaction mixture, triethanolamine (TEOA) was also present to act as a sacrificial electron donor and proton source. During each reaction, BrAPN, TEOA, and MO_x_‐**Ir** (0.2 mol %) were added to a vial with deuterated acetonitrile (CD_3_CN) and a stir bar. The reaction was sealed and degassed with Ar prior to measurement and kept as dark as possible (more reaction details in Supporting Information). Reactions were stirred rapidly at 1000 RPM, and illuminated with a blue Kessil LED with a 435 long pass filter, placed precisely 3 cm away from the vial (∼125 mW/cm^2^); the reaction was also kept cool with a fan blowing on it at all times. These conditions were kept constant to minimize error between measurements (extra details in Supporting Information).

The photoredox catalytic reactions were initially examined using the nanopowder catalysts over two hours of reaction time (Table 2, entry 1–3). Reactions were performed in CD_3_CN in order to analyze the reaction mixture by ^1^H NMR spectroscopy (details in Supporting Information). Importantly, a main advantage of this reaction is that it is easily followed by ^1^H NMR as BrAPN and APN have singlets in non‐overlapping regions; BrAPN has a singlet at 4.68 ppm for its CH_2_ while APN has a singlet at 2.56 ppm for its CH_3_ (Figure S7). Amazingly, all nanopowder catalysts showed full conversion to APN after two hours of reaction.

**Table 2:** Reaction yields for reductive dehalogenation of bromoacetophenone to acetophenone with MO_x_‐Ir nanopowders and control reactions after two hours.

Entry	Sample	Light / Dark	% Yield APN
1	ITO‐Ir	Light	100
2	Al_2_O_3_‐Ir	Light	100
3	ZrO_2_‐Ir	Light	100
4	ITO	Light	0
5	Al_2_O_3_	Light	4
6	ZrO_2_	Light	2
7	ITO‐Ir	Dark	0
8	Al_2_O_3_‐Ir	Dark	0
9	ZrO_2_‐Ir	Dark	0
10^[a]^	ITO‐Ir	Light	23
11^[a]^	Al_2_O_3_‐Ir	Light	25
12^[a]^	ZrO_2_‐Ir	Light	20
13^[b]^	ITO‐Ir	Light	0
14^[b]^	Al_2_O_3_‐Ir	Light	0
15^[b]^	ZrO_2_‐Ir	Light	0
16	No catalyst	Light	0
17^[c]^	No catalyst	Light	16

[a] Reactions used a ratio of BrAPN:TEOA:MO_x_‐Ir of 1:0.5:0.002; [b] 0 equivalents of triethanolamine in reaction; [c] reaction performed without 435 nm long pass filter.

### Reaction controls

Since the reactions showed all catalysts were functional, several controls were then performed to make sure catalysis was occurring as expected (Table 2). First, we tested to ensure the catalyst was necessary for the reaction to occur (entry 16). No APN was formed when the catalysts were removed from the reaction mixture, suggesting they were necessary for catalysis. To ensure that the molecular catalyst was necessary, controls were performed with just the metal oxide nanopowders (without catalyst bound) in the reaction (entry 4–6). Insignificant APN was produced, suggesting that the molecular component is necessary for photoredox catalysis. Furthermore, the reactions do not work in the dark, suggesting that light is necessary to excite the catalyst and promote turn over (entry 7–9). In addition, the necessity for triethanolamine was tested. No APN was formed without TEOA present (entry 13–15), as this is likely needed to i.) reduce the iridium excited state, Ir*, and ii.) donate a proton to the reduced BrAPN species, which forms the APN product. When the concentration of TEOA is half that of BrAPN (entry 10–12), reaction yields are only 20–25 %, suggesting there is not enough reagent around to finish the catalytic reaction. Finally, when the concentration of TEOA is three times that of BrAPN (entry 1–3), reactions yields go to completion. This trend suggests that we do need TEOA in the reaction mixture and in excess to the BrAPN substrate.

**Figure 4:**
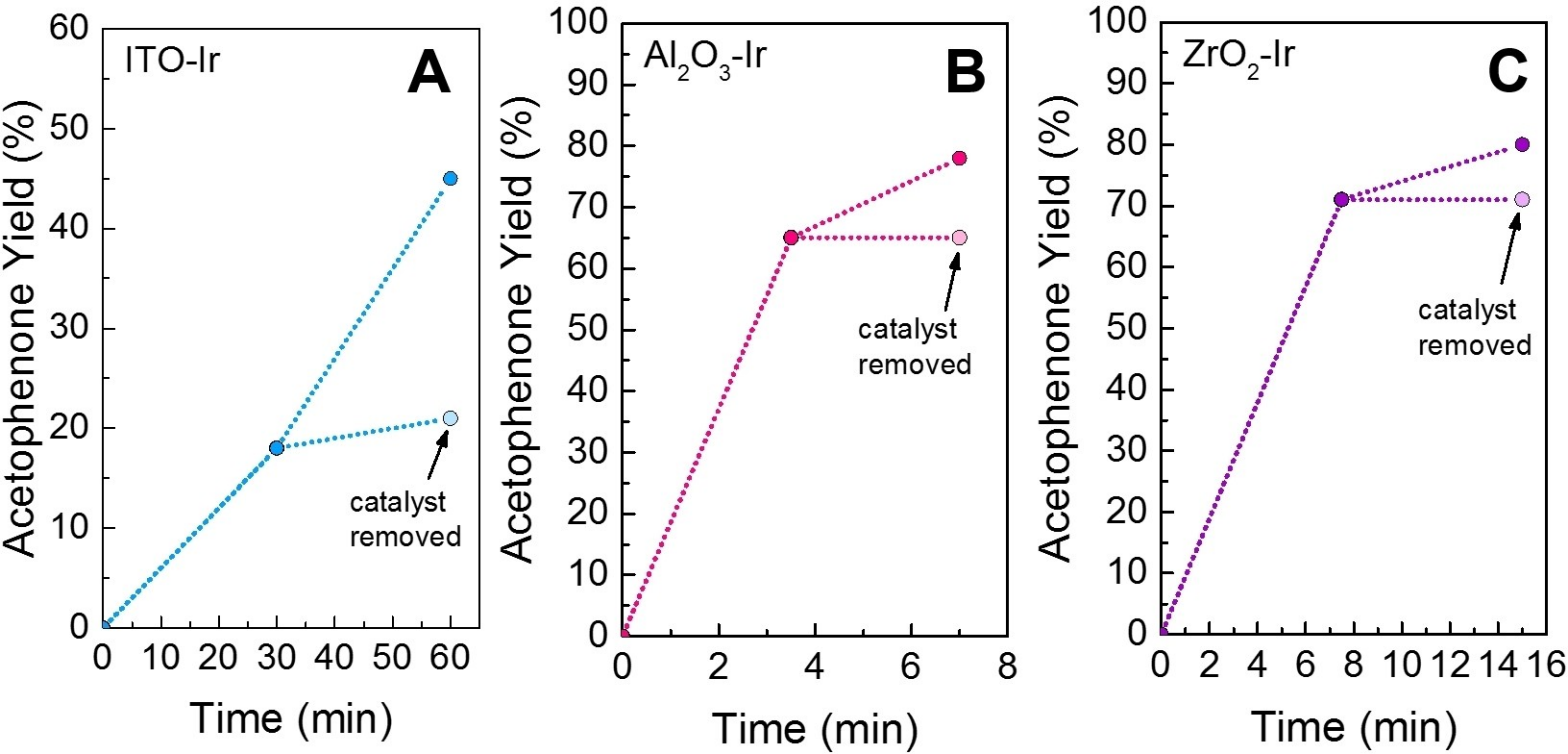
Heterogenized tests for (A) ITO−Ir (blue) (B) Al_2_O_3_‐Ir (pink), (C) ZrO_2_‐Ir (purple). Reactions were run for a few minutes, catalyst removed from the reaction mixture and yields quantified. The reaction was continued without the catalyst for a few more minutes, and the yield checked again (light colored circle). This was compared to when the catalyst was added in the reaction for the same amount of time.

Finally, as there is always a possibility that catalyst desorption can occur in heterogenized systems,^[8]^ we wanted to check that the active catalyst was indeed heterogenized and not a desorbed homogeneous catalyst. To do this, the reaction yield was analyzed after a few minutes (to ensure the reaction was not complete), catalyst removed from the mixture, and APN yield quantified. The reaction was then continued without the catalyst present to see if the yields changed when the catalyst was removed. If the yield increased, this would suggest that the active catalyst was actually a desorbed **Ir** species and not the heterogenized catalyst. If the yield did not change, then the active catalyst is likely heterogenized. Indeed, for all catalysts the yield did not increase significantly, suggesting that the active catalyst was heterogenized (Figure 4). Moreover, if the catalyst was added back into the reaction, yields increased again, further confirming that the catalyst was heterogenized (Figure 4).

### Mechanistic insight

**Figure 5:**
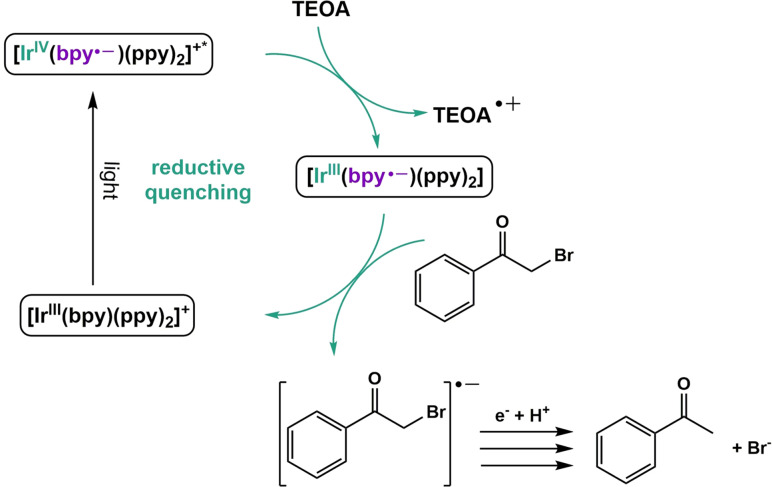
Proposed catalytic cycle based on TCSPC data and electrochemical data. First, **Ir** (depicted more precisely as [Ir^III^(bpy)(ppy)_2_]^+^, where ppy=2‐phenylpyridine, and bpy=2,2′‐bipyridine‐4,4′‐dicarboxylic acid) becomes photoexcited upon illumination, forming the excited iridium species^[6]^, which is then reduced by TEOA to form the reduced iridium state. Finally, the reduced iridium species reduces BrAPN, which returns **Ir** to its ground state. The reduced BrAPN then goes on to form the final product after subsequent reduction and protonation via reaction with the triethanolamine radical cation.^[25]^

Since the controls suggested that the catalyst was functioning as a heterogenized catalyst, we postulated what mechanistically could be occurring in the reaction mixture. Based on the above controls and electrochemical data, we proposed that upon illumination i.) **Ir** becomes photoexcited forming the excited state species ii). the excited state species is reduced by TEOA forming the reduced iridium catalyst, and the reduced catalyst reduces the BrAPN substrate, which goes on to form the product through subsequent reduction and protonation steps (Figure 5). To further support this hypothesis, TCSPC measurements were performed on **Ir** in the presence of TEOA, which showed evidence for excited state quenching; this is seen in the decrease in the excited state lifetimes from 210 ns to 80 ns in the presence of TEOA (Figure S35). The excited state decay of **Ir** shows a much smaller change in the presence of BrAPN, with a lifetime of 180 ns (Figure S35, Table S5). Concentrations of the **Ir**, BrAPN, and TEOA were kept the same as the reaction conditions during the TCSPC measurements to keep conditions as realistic as possible. These additional experiments suggest that in the catalytic cycle, excited **Ir** is first reductively quenched, which is then followed by BrAPN reduction as shown in Figure 5. Recall from the electrochemical and photophysical data that the reduced state of **Ir** is thermodynamically capable of reducing BrAPN, while the excited Ir* is not, further supporting the cycle in Figure 5. We note that these TCSPC experiment were done in the homogeneous phase for simplicity, and mechanistic results could change in the heterogenized system. This will be evaluated more extensively in a later study.

### The need for speed

**Figure 6:**
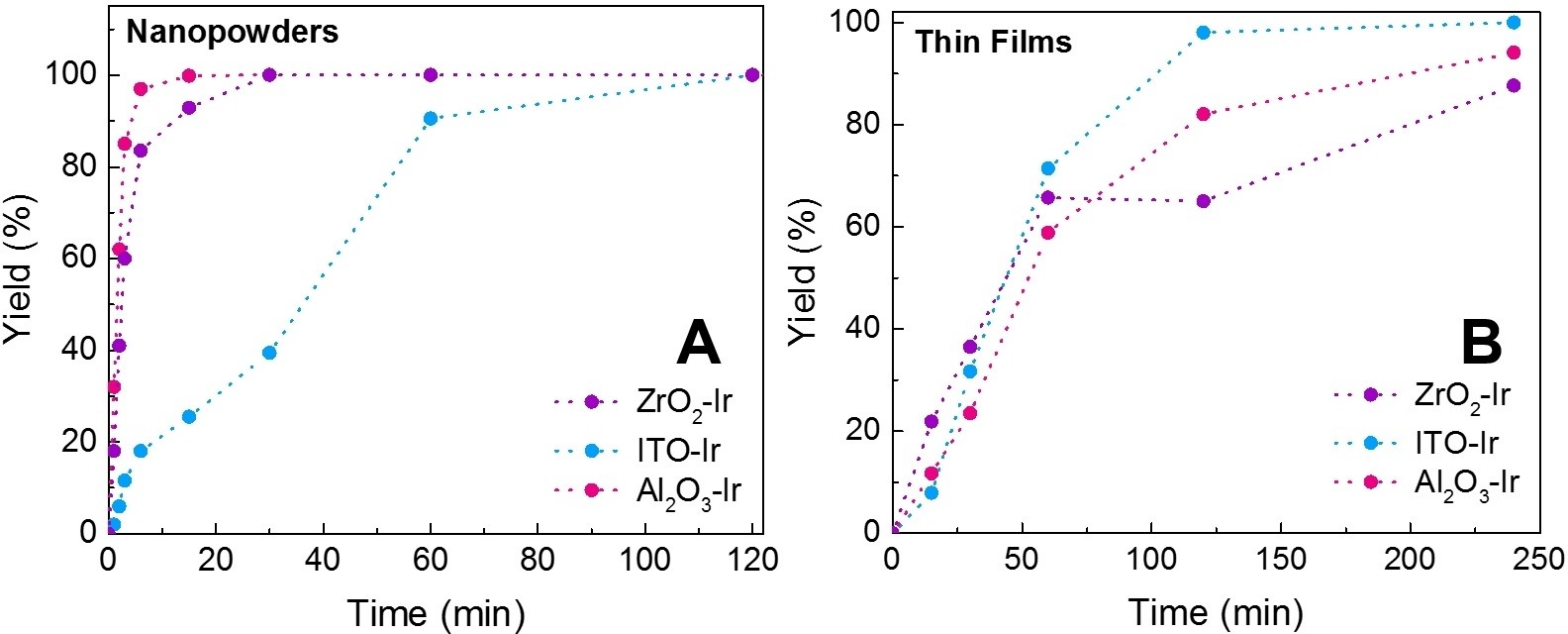
Reaction yield over time during photoredox catalysis using (A) nanopowder and (B) thin film catalysts (ITO‐**Ir** (blue), Al_2_O_3_‐**Ir** (pink), and ZrO_2_‐**Ir** (purple)).

With a grasp of what could be happening during catalysis, and as the reactions reached completion during two hours, we wondered if the reactions were complete prior to the two‐hour mark. To do this, we tracked the reaction over time by ^1^H NMR and found that indeed, the nanopowder‐based catalysts were remarkably faster (Figure 6A). For example, Al_2_O_3_‐**Ir** samples showed the fastest APN formation with reactions complete by 15 minutes doing 385 turnovers (limited by substrate consumption) with a turnover frequency (TOF) of ∼0.4 s^−1^. ZrO_2_‐**Ir** samples were also fast, reaching completion after 30 minutes, being slightly slower with a TOF of ∼0.2 s^−1^. ITO‐**Ir** was the slowest, taking the full two hours to reach completion giving it a TOF of 0.05 s^−1^. Clearly, the fastest catalysts are those using wide bandgap semiconductor supports, which suggests these are the optimal surfaces for the nanopowder reactions. ITO supports may have resulted in slower reactions because the **Ir** excited state lifetime is much shorter on these surfaces, or if the surface is non‐innocent during catalysis (e. g. from electron injection into ITO by Ir* or **Ir^−^**).[^2h^, ^21^] Table 3 summarizes these findings.

**Table 3:** Reaction times, TOFs, TONs for the nanopowder and film catalysts.

Nanopowder Catalysts	Reaction Time (min)^[a]^	TOF (s^−1^)	TON (post 3x uses)
Al_2_O_3_‐Ir	15	0.4	942^[b]^
ZrO_2_‐Ir	30	0.2	663
ITO‐Ir	120	0.05	777^[b]^

Film Catalysts	Reaction Time (min)^[a]^	TOF (s^−1^)	TON (post 3x uses)
Al_2_O_3_‐Ir	240	0.025	984^[b]^
ZrO_2_‐Ir	240	0.025	798
ITO‐Ir	120	0.05	558

[a] Time for the reaction to reach completion; [b] More turnovers possible.

### Thin film reactions

Since the nanopowder reactions were highly functional using heterogenized iridium photoredox catalysts, we wanted to test if thin films could also be used for these reactions as they are easier to characterize, easier to remove from the reaction, and have possible applications in future (photo)electrochemical setups. Reactions were performed with the thin films placed in the reaction vial, face up, to prevent the stir bar from removing the thin film (see Figure S3). The equivalents of the reagents (BrAPN, TEOA, and **Ir**) in the thin film and nanopowder reactions were kept constant in order to compare the two systems, and the time needed to reach reaction completion was monitored by ^1^H NMR (Table 3). Since there is no catalyst diffusion or transport via stirring with thin films, we expected the reactions to take longer than the nanopowders. Indeed, reactions for both Al_2_O_3_‐**Ir** and ZrO_2_‐**Ir** films were much slower (Figure 6B), taking four hours to reach completion and perform 385 turnovers, giving them a TOF of 0.025 s^−1^. Interestingly, ITO‐**Ir** remained the same, taking 2 h still to complete. Since ITO‐**Ir** nanopowders and films took similar reaction times, this suggests that ITO may not be acting solely as a catalyst platform; perhaps electron transfer events between the catalyst and ITO (such as electron injection) are also occurring during these reactions, which can alter the reaction times.

We note that the catalyst concentration in the reaction mixture was lower with the thin films, which may contribute to the slower reaction times. However, the effect of the catalyst architecture (nanopowder versus film) on reaction completion time is likely more significant as the nanopowder reactions for Al_2_O_3_‐Ir are complete sixteen times faster than the films, even though the catalyst concentrations are only approximately four times lower. This suggests that the thin film architecture is likely contributing more to the slower catalysis than the catalyst concentration.

### Comparison to homogeneous Ir

Finally, we were curious how our photoredox catalysts compared to **Ir** in a homogenous system. When this reaction was followed, we found it was quite similar to the ZrO_2_‐**Ir** nanopowder system taking 30 minutes to reach completion (Figure S5). Interestingly, the fastest nanoparticle‐based heterogenized catalyst, Al_2_O_3_‐**Ir**, operates more efficiently than the homogeneous catalyst. This is noteworthy as Al_2_O_3_‐**Ir** also has the advantage of being easier to separate from the reaction mixture, making it the more reusable option.

### Catalyst integrity

**Figure 7:**
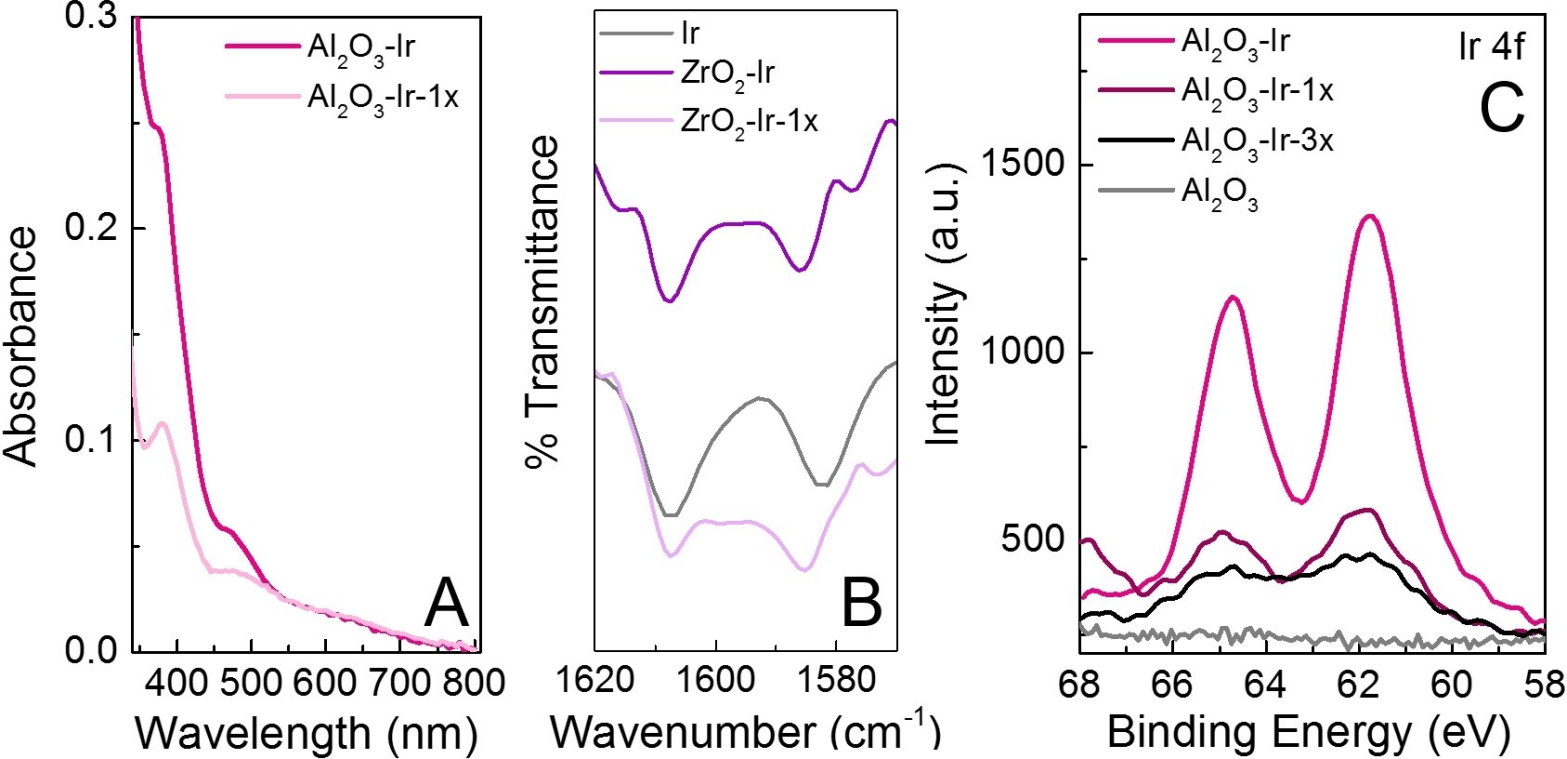
(A) UV‐Vis spectra of Al_2_O_3_‐**Ir** before (dark pink) and after (light pink) one photoredox catalytic test. (B) ATR‐FTIR spectra of ZrO_2_‐**Ir** before (dark purple) and after (light purple) one photoredox catalytic test; **Ir** powder is shown in grey. (C) XPS spectra of Al_2_O_3_‐**Ir** (dark pink) and Al_2_O_3_ blank (grey). The XPS spectra of Al_2_O_3_‐**Ir** after one photoredox test is shown in red and after three tests is shown in black.

Since both nanopowder and film catalysts showed excellent photoredox activity, we wanted to check the catalyst integrity after reactions to see if they could be good candidates for reusability measurements. UV‐Vis of the thin films showed that **Ir** was retained on the surface (Figure 7A, S13). ATR‐FTIR of the catalysts also indicated that the C=C and C=N stretches were retained (Figure 7B, S22‐23), and XPS showed that iridium remained on the metal oxide supports (Figure 7C, S28–29). These experiments suggest retention of the **Ir** structure on the surface and that they have the potential to be reusable.

### Catalyst reusability

**Figure 8:**
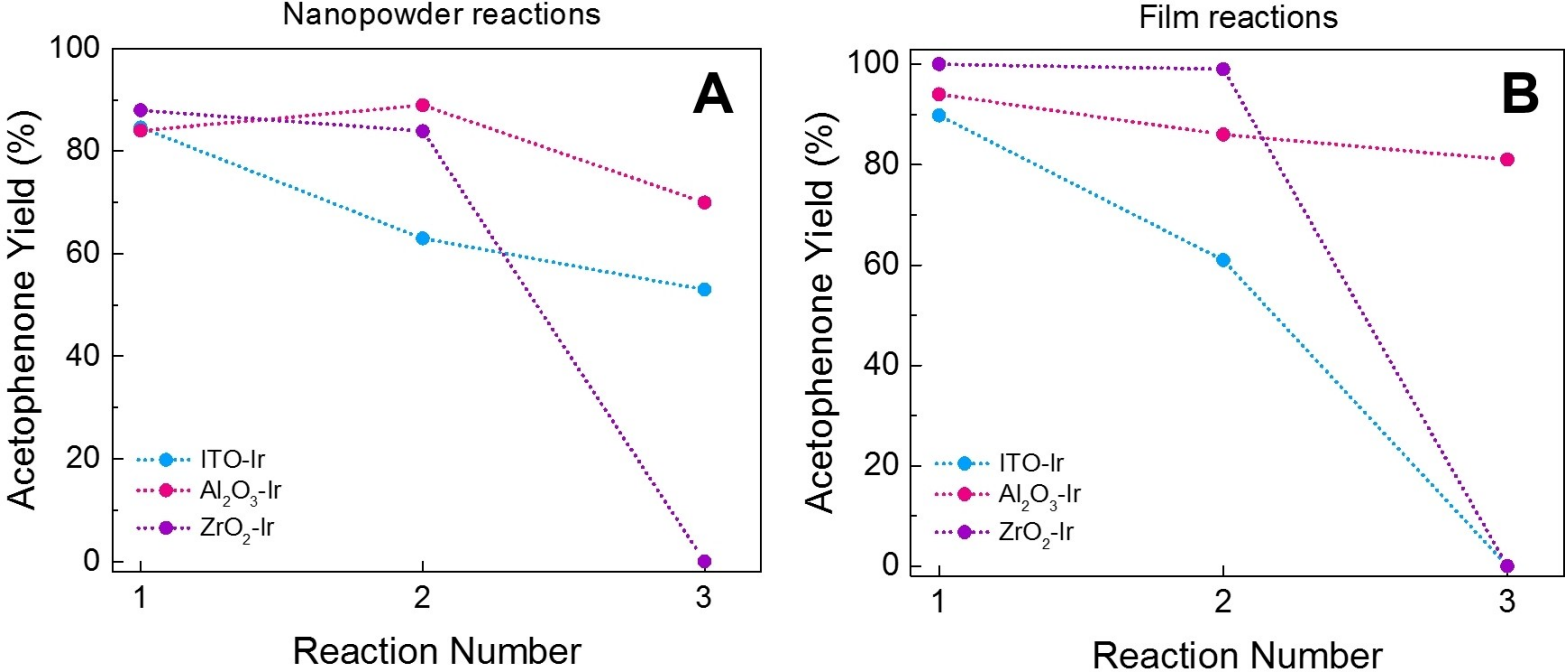
Reaction yields for three sequential photoredox tests for (A) nanopowder catalysts and (B) thin film catalysts; ITO‐**Ir** (blue), Al_2_O_3_‐**Ir** (pink), ZrO_2_‐**Ir** (purple).

Since **Ir** remained mostly surface‐bound after catalysis, reusability experiments were performed to see if the catalysts could be used more than once (Figure 8, Table 3). All catalysts showed good yields over two uses, with yields only dropping slightly to ∼90 % for Al_2_O_3_‐**Ir** and ZrO_2_‐**Ir** catalysts. ITO‐**Ir** saw a greater drop after two uses to around ∼60 % yield (Figure 8). After three uses, ZrO_2_‐**Ir** nanopowder and film catalysts as well as ITO‐**Ir** nanopowders did not produce APN, suggesting that they had reached maximum turnover; ZrO_2_‐**Ir** nanopowder performed 663 turnovers, ZrO_2_‐**Ir** films 798 turnovers, and ITO‐**Ir** films 588 turnovers prior to losing activity. ITO‐**Ir** nanopowders continued to function after three uses, dropping steadily in yield over the uses to 60 % yield by the end of the third reaction performing 777 turnovers as tested (more possible). The best catalysts for reusability in both films and nanopowders were those of Al_2_O_3_‐**Ir** films, with yields staying above ∼80 % yield over three uses, and just at ∼80 % for the nanopowders. Clearly, these are the most robust metal oxides for this reaction, and conveniently the fastest catalysts in the nanopowder form. After three uses, Al_2_O_3_‐**Ir** films performed 984 turnovers and nanopowders 942 turnovers, with more turnovers possible as they may be able to be reused further. Table 3 summarizes the TONs from the reusability tests.

Furthermore, Al_2_O_3_ and ITO films were the most robust supports, as no metal oxide film loss was observed after the reactions, unlike ZrO_2_ films, which peeled away under the reaction conditions, likely limiting the reusability. Nanopowder reactions, although faster, do lose some of the initial MO_x_‐**Ir** starting mass after each use due to losses during centrifugation and rinsing steps, even up to 50 % of the initial catalyst mass by the start of reaction three (Figure S6). Clearly, as the Al_2_O_3_ and ITO films are retained after each use, these are the most robust and easily reusable platforms. Regardless, as all catalysts were reusable at least twice, this highlights that heterogenized catalysts have the potential to be used as an environmentally friendly alternative way to do photoredox catalysis when fully optimized.

### Catalyst integrity after reusability

To understand why some of the catalysts were not as reusable, they were characterized again after three uses. Characterization of the film samples by UV‐Vis showed catalyst loss or degradation after each use with **Ir** signature bands decreasing after each use (Figure S14), and similarly when ATR‐FTIR was used (Figure S24, 25). XPS still showed iridium content on most of the metal oxides, demonstrating that the catalyst had not fully desorbed (Figure S30, 31). Based on these results, some of the iridium complex has been removed from the metal oxide surface after three uses, which could be a reason for the loss in activity with some catalysts.

### Reaction mixtures

To further understand what was happening during catalysis, we examined the UV‐Vis spectra of the reaction mixtures post catalysis during the reusability tests. The UV‐Vis spectra of the reaction mixtures after the first two reactions showed a species present in the UV‐Vis spectra (Figure S15, 16). The species observed differed depending on what catalyst was used, and could be either a desorbed, degraded, or reduced **Ir** species. Note that the spectra did not match that of **Ir** for nearly all of the catalysts with the exception being from the ITO‐**Ir** nanopowder reactions where the UV‐Vis spectra from the first reaction appeared to mimic **Ir**, suggesting some **Ir** desorption in this specific case.

To understand the origin of these species, we tested to see if they were formed before or during catalysis. First, controls were performed to examine if the species was formed prior to catalysis by soaking the MO_x_‐**Ir** films in the dark in the presence of acetonitrile solutions of BrAPN, TEOA, a mixture of both (Figure S18). Recall, that solvent and light alone, does not desorb **Ir** (Figure S17). We found that a species desorbs when films are exposed to both BrAPN and TEOA prior to catalytic measurements (prior to LED exposure), which has absorption bands around 400 nm and 500 nm (Figure S18F); this spectrum is similar to that of **Ir**, but has shifted slightly, which could suggest some changes in the ligand environment of the iridium complex due to exposure to both BrAPN and TEOA. This suggests that prior to illumination, some desorbed/altered iridium species is already present in the reaction mixture. However, these spectra do not match that of the reaction mixtures post catalysis, suggesting that i) the species that desorbs prior to catalysis may change some during photoredox catalysis or ii) that another degraded/changed species desorbs from the metal oxide surface during catalysis. We note that soaking the catalyst in an APN acetonitrile mixture does not match these spectra either, suggesting that APN does not aid in forming the species. For now, it is clear that an iridium species is appearing in the reaction mixtures, but it is different from **Ir**. Importantly, recall that when the catalyst is removed from the reaction mixture during catalysis (Figure 4), the reaction mixture alone does not show catalytic activity. Thus, we conclude that these desorbed/degraded species are likely innocent during catalysis as they are possibly i) an inactive complex slightly different than **Ir** or ii) too low in concentration to contribute to catalysis. Nonetheless, loss of the surface species or degradation is likely a reason for some loss in catalytic activity for some of the samples, and will be optimized and investigated further in future work.

## Conclusions

In this study, we have performed a systematic study to analyze the effect of the catalyst support in heterogenized iridium photoredox catalysts. Three metal oxide supports were examined in both nanopowder and thin film form, and all catalysts were found to be functional for reductive dehalogenation reactions to form acetophenone. Significantly, the fastest catalyst, nanopowder Al_2_O_3_‐**Ir**, was able to reach reaction completion in nearly 15 minutes, which is slightly faster than the homogenous system. Thin film‐based catalysts also gave high yields, but operated slower (2–4 h) than their nanopowder counterparts due to no catalyst diffusion or transport via stirring. However, thin films provide more applicable architectures in future (photo)electrochemical photoredox catalytic studies and additionally, were more easily reused. Post catalysis, all catalysts showed good surface stability and were able to be reused at least 2–3 times. The most reusable catalysts were Al_2_O_3_‐**Ir** (both film and nanopowder) showing a limited drop in yield after recycling, which approached 1000 turnovers after three uses, further highlighting that these catalysts can be used to do efficient photoredox catalysis sustainably. Thus, of the three surfaces, Al_2_O_3_ is the optimal metal oxide, and thin film supports are preferred over the nanopowders as they are the most easily reused and greener options; in future work, catalysts will be further explored with these architectures. Since some surface instability was observed during the reactions, catalyst stability on the metal oxide will be improved in future investigations by using stronger surface anchors or through the addition of surface protecting layers. We are currently working to expand the substrate and reaction scope with the optimized catalysts, to show broader applicability in future studies. Finally, we hope to have shown here that heterogenized catalysts have a great potential to be used in the photoredox community, bringing together both the tunability of homogeneous systems and reusability of heterogeneous systems in one complete, environmentally friendly catalyst.

## Experimental Section

Further experimental details on catalyst preparation and characterizations, reaction conditions, and NMR quantifications can be found in the Supporting Information. Additional UV‐Vis, ATR‐FTIR, photoluminescence, XPS, and NMR spectra and TCSPC results can also be found in the Supporting Information.

## Conflict of interest

The authors declare no conflict of interest.

## Supporting information

As a service to our authors and readers, this journal provides supporting information supplied by the authors. Such materials are peer reviewed and may be re‐organized for online delivery, but are not copy‐edited or typeset. Technical support issues arising from supporting information (other than missing files) should be addressed to the authors.

Supporting Information
