# Supplementary material for: Photoredox Catalysis Using Heterogenized Iridium Complexes
Source: Chemistry. 2021 Jul 22;27(68):16966–77. doi: 10.1002/chem.202101651 (PMC9292873; doi:10.1002/chem.202101651)
Supplement: Supplementary file 1 — Supporting Information [file CHEM-27-16966-s001.pdf]

Chemistry–A European Journal

Supporting Information

Photoredox Catalysis Using Heterogenized Iridium Complexes

Kelly L. Materna* and Leif Hammarström

Experimental

Instrumentation. A Varian Cary-50 Bio spectrophotometer was used to perform UV-Vis spectroscopic measurements. A Bruker IFS 66/S FT-IR spectrometer with an ATR accessory was used to perform the ATR-FTIR measurements. A Horiba Fluorolog®-3 fluorimeter was used to perform photoluminescence measurements. X-ray photoelectron spectroscopic measurements were performed using a A PHI Quantera SXM using a monochromatic Al Kα source (hν = 1486.6 eV) operated at 25 W with a beam diameter of 100 μM. Survey spectra were collected with a pass energy of 224 eV and high-resolution spectra with a pass energy of 55 eV. Data were analyzed using Multipak software and all spectra calibrated by setting adventitious carbon to 284.8 eV. A home-built experimental setup was used to perform time-correlated single photon counting (TCSPC) measurements as previously described.[1] An Autolab potentiostat was used to perform the electrochemical measurements. A JEOL (400YH magnet) Resonance 400 MHz spectrometer was used to collect the ^{1}H NMR spectra; chemical shifts are reported (δ) and are referenced to the residual proton solvent signal.

Reagents and Materials. Indium tin oxide (ITO) nanopowder (<50 nm), aluminum oxide nanopowder (<50 nm), and zirconium(IV) oxide (<100 nm) were purchased from Merck and used for the nanopowder-based catalysts. Al-Nanoxide A/SP and Zr-Nanoxide ZT/SP paste were purchased from Solaronix to prepare the thin film samples. All other reagents and solvents were purchased from Merck unless otherwise stated.

Synthesis of Ir. Ir was synthesized identically to a previously reported procedure[2] (Scheme S1). Briefly, 108 mg (0.1 mmol) of di-μ-chlorotetrakis[2-(2-pyridinyl-kN)phenyl-kC]diiridium(III) ([Ir(ppy)$_2$(μ-Cl)]$_2$) was weighed out and dissolved in a dichloromethane solution via sonication, forming a yellow-olive colored solution. Separately, 50 mg (0.2 mmol) of 2,2′-bipyridine-4,4′-dicarboxylic acid was added to 10 mL of methanol, forming an insoluble white mixture. The Ir/dichloromethane solution was then added dropwise to the bipyridine/ methanol mixture over one minute, forming a yellow solution. The reaction was heated and attached to a reflux condenser. The reaction was gently refluxed (46 ^{o}C) for two hours forming a yellow/orange mixture. Afterwards, 2.0 mL of excess (8 equiv.) sodium acetate (NaOAc) in methanol was added to the reaction and heated for one hour further. During this time, the reaction turned from green to orange with increasing solubility. The reaction was then cooled to room temperature, 2.0 mL of saturated ammonium hexafluorophosphate (NH_4PF_6) in methanol was added, and stirred for 30 minutes forming a clear orange/red solution. The solvent was rotary evaporated off, affording an orange/white solid. Next, 10 mL of 1 M HCl (aq) was added to the solid and was stirred for 10 minutes with some sonication to remove solid from sides of the flask. The solid was filtered off and washed with 2 x 10 mL water. The solid was extracted into 40-50 mL of methanol, 2 mL of saturated NH_4PF_6 in methanol was added, and the mixture was stirred for 30 minutes. The solvent was removed under vacuum and the solid suspended in dichloromethane, forming a dark red cloudy solution. A solid was filtered off from the dark red mixture and rinsed with dichloromethane multiple times, affording a bright orange solid. The solid was dried under vacuum (54.1 mg, 30% yield). ^{1}H NMR (400 MHz, d_6-DMSO): δ 9.16 ppm (s) 2H, 8.26 ppm (d, J = 8.1 Hz) 2H, 8.01 ppm (dd, J = 21.1, 5.7 Hz) 4H, 7.96 – 7.89 ppm (m) 4H, 7.64 ppm (d, J = 5.7 Hz) 2H, 7.12 ppm (t, J = 6.6 Hz) 2H, 7.02 ppm (t, J = 7.5 Hz) 2H, 6.90 ppm (t, J = 7.4 Hz) 2H, 6.16 ppm (d, J = 7.5 Hz) 2H.

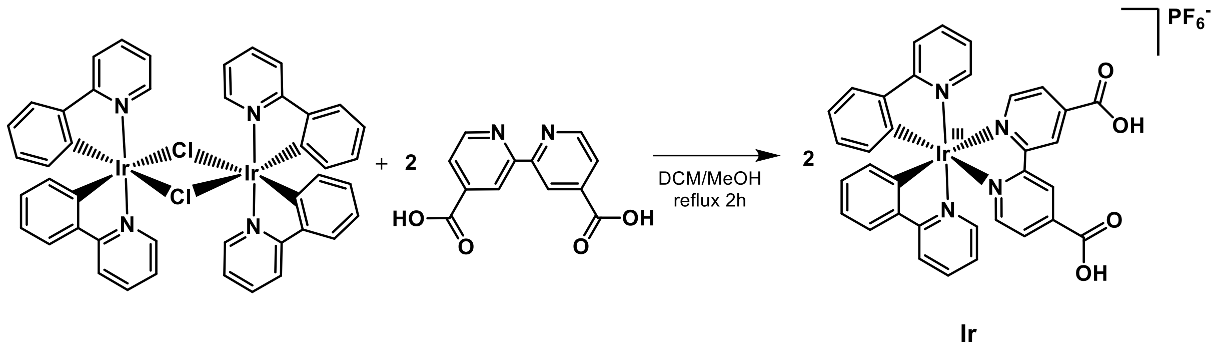

Scheme S1. Synthesis of **Ir**.

Preparation of Thin films. To prepare the different ITO, Al_2O_3, and ZrO_2 thin film platforms, a doctor blading method was used followed by annealing at high temperatures. For the doctor blading procedure, metal oxide pastes were needed. Al_2O_3 and ZrO_2 pastes were used as purchased from Solaronix and the annealing instructions followed via Solaronix´s guidelines. ITO paste was prepared similar to prior work[3] by sonicating and simultaneously stirring 0.5 g of ITO nanopowder with 0.5 g of glacial acetic acid for five minutes; 1.0 mL of ethanol was added to this mixture and was sonicated and stirred for five minutes, creating a green liquid/paste. Next, FTO coated glass slides (TEC15) were cleaned via subsequent sonication in water, acetone, and methanol. The glass slides were then masked with Scotch Magic™ tape on the top edge and two side edges leaving space in the middle for the paste. The glass slides were then wiped with methanol once more to clean the exposed glass surface once more prior to doctor blading. When the surface was dry, one layer of the metal oxide paste was doctor-bladed onto the glass slides by placing a small drop of the paste on the tape at the top of the glass slide and then spreading the paste from top to bottom using a glass rod. The tape was then carefully removed, and the glass slides were sintered face-up on a hotplate at 120 ^{o}C for 10 minutes. All thin films were then annealed face-up at higher temperatures in an oven; ITO films were annealed at 500 ^{o}C for one hour, Al_2O_3 films at 400 ^{o}C for 30 minutes, and ZrO_2 films at 500 ^{o}C for 30 minutes. Post annealing, film thicknesses were measured using a profilometer. Film thicknesses were are follows: 1.7 µm for ITO, 2.8 µm for Al_2O_3, and 4 µm ZrO_2.

Catalyst Loadings. To obtain the catalyst loadings, two methods were used. For the thin film samples, UV-Vis spectra were recorded directly on the thin films (Figure 3). The loadings were then determined using the formula $\Gamma(\text{mol cm}^{-2}) = A(\lambda)/(1000\varepsilon)$, where A is the absorbance at wavelength λ, and ε is the molar extinction coefficient at wavelength λ.[4] **Ir** has a signature peak at 370 nm and was used for these calculations ($\varepsilon = 8737$ M^{-1} cm^{-1}, Figure S10). Average loadings can be found in Table S2.

For the nanopowder catalysts, the depletion method was used as previously described.[3] Briefly, the UV-Vis spectrum of the **Ir** sensitization solution was recorded before and after sensitizing the nanopowders. The concentration of **Ir** was then calculated from these spectra using $A(\lambda) = \varepsilon bc$, where $A(\lambda)$ is the absorbance at wavelength λ, ε is the molar extinction coefficient at that wavelength, b is the path length, and c is the concentration; the peak at 370 nm was used for these calculations. A decrease in **Ir** concentration after sensitization was was observed (Figure S12), suggesting that the **Ir** that was in the sensitization solution was likely

removed from the solution via surface binding to the metal oxide supports. Average loadings can be found in Table S1.

Table S1. Average loadings of nanopowder catalysts.

**Nanopowders**	**Average Loading (nmol /mg)**
ITO-**Ir**	6.7 ± 0.9
Al_2O_3-**Ir**	6.0 ± 0.9
ZrO_2-**Ir**	7.1 ± 0.8

Table S2. Average loadings of thin film catalysts.

**Thin Films**	**Loading (nmol /cm^2)**
ITO-**Ir**	35.8 ± 7.0
Al_2O_3-**Ir**	28.4 ± 6.9
ZrO_2-**Ir**	31.1 ± 8.7

Electrochemistry. For cyclic voltammetric measurements, a one-pot air-tight cell was used. A three-electrode setup was used incorporating a pseudo Ag/AgCl reference electrode, glassy carbon working electrode, and platinum wire auxiliary electrode; ferrocene was added at the end of each measurement as an internal standard. A 0.1 M $TBAPF_6$ electrolyte in acetonitrile was used, and all measurements were performed under Ar.

F. Photoredox catalysis details

Reaction setup details: For each reaction, conditions were kept as similar as possible to minimize error. The reactions were performed in 5.0 mL microwave reaction vials in order to provide an air-tight seal. For all reactions, stir bar size was kept constant and stirring rate kept at 1000 RPM. Prior to catalysis, all reactions were degassed with high purity Ar from a gas cylinder, and the lids further protected with putty to seal the needle holes from the degassing procedure. The vials were placed on a stir plate 3 cm from a Kessil A160we- tuna blue LED lamp with a 435 nm long pass filter (~125 mW/cm^2). A filter was used to prevent bromoacetophenone excitation as the lamps absorption overlaps with the substrate (Figure S1). A fan was also placed near the vial and used to prevent heating of the reaction due to the LED. Figure S2 shows a picture of an example of the reaction setup described above.

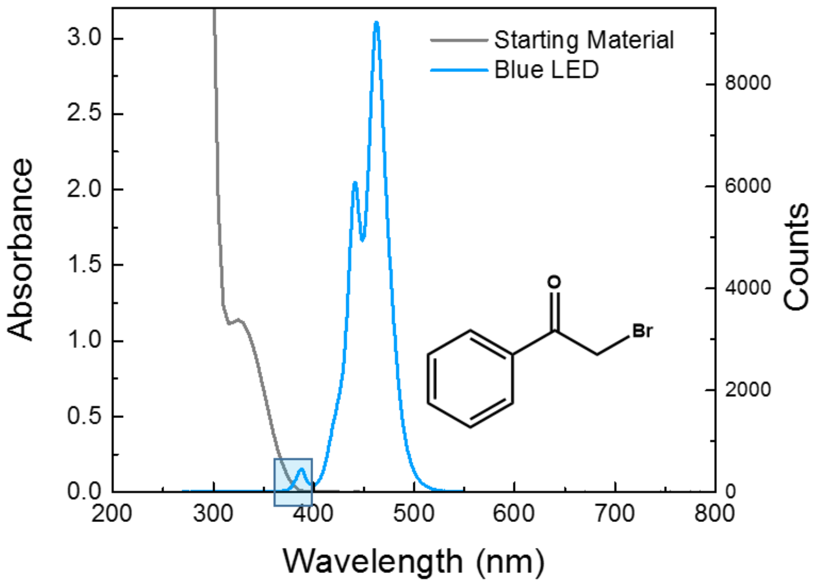

Figure S1. UV-Vis spectrum of bromoacetophenone (grey). The trace in blue shows the spectrum collected from the Blue Kessil LED using a photodiode. Clearly, there is some overlap between the two spectra, indicating the lamp can photoexcite bromoacetophenone. In fact, upon excitation, bromoacetophenone will form acetophenone (without catalyst) if the long pass filter is not used (Table 2, entry 17).[5] Thus, a long pass filter is used in the experiments to prevent this and only excite the catalyst, allowing the effect of the catalyst to be investigated.

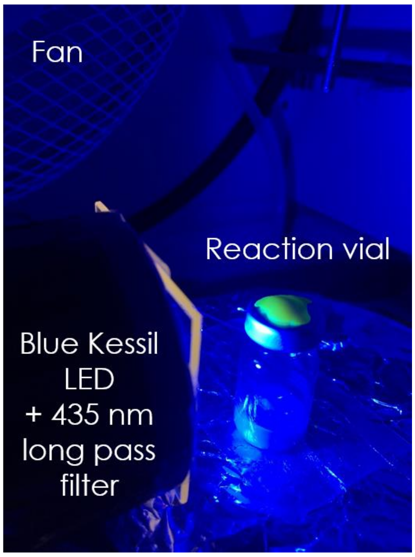

Figure S2. Photo of reaction setup. A sealed microwave reaction vial is placed 3 cm from a Blue Kessil LED with a 435 nm long pass filter. A fan is also turned on during the reactions to cool this vial and avoid temperature changes.

Thin film reaction considerations. We note that for the thin film reactions, correct orientation of the thin film in the reaction and optimal stir bar size was important for film stability. Figure S3 shows what the films look like after stirring in acetonitrile for 24 hours when the films are face down, face up, and face down with a gap at the bottom for three different stir bar sizes. If they are face down, the stir bar can sometimes bump the film and cause film loss. Even with a gap at the bottom, film loss is still observed when the films are face down. When the films are face up, the thin film is retained on the glass slide. We chose to examine several stir bar sizes since we wanted to choose one that would keep the film intact but also stir the reaction effectively (larger being preferred). For the films face down, all stir bars caused loss of the film, with the smallest round stir bar providing the least film loss and the largest one providing the most loss. When there is a gap at the bottom of the slide and the films are face down, again, more film loss is observed as the stir bar size is increased. For the face up films, the thin film was retained regardless of the stir bar size. Thus, reactions were performed with the films face up in the with the stir bar orientated as shown in Figure S3.

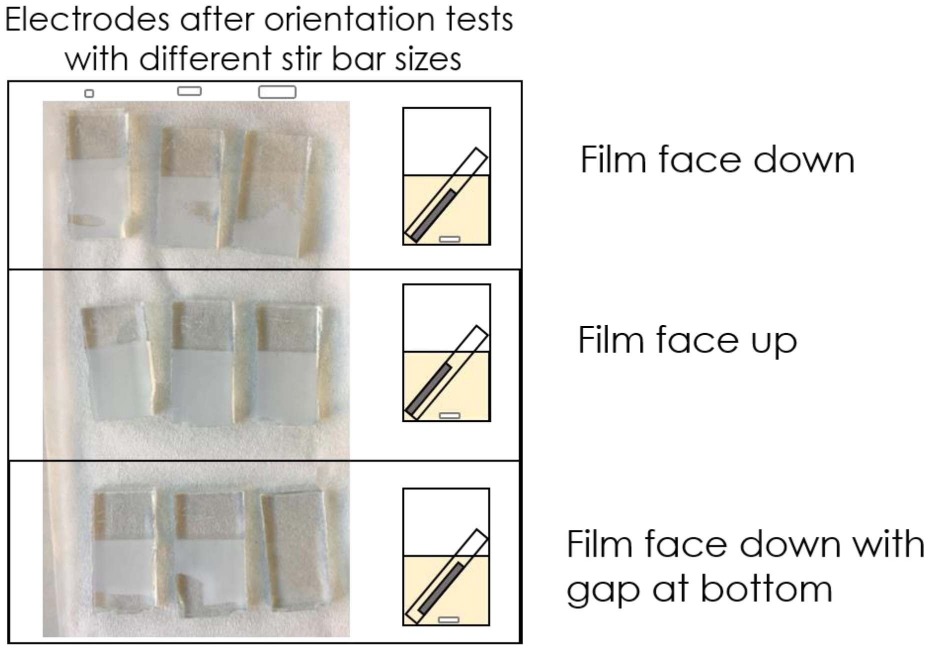

Figure S3. Thin films of ZrO_2 after orientation and stir bar size tests. The films were stirred in acetonitrile for 24 hours and the resulting films are shown above. The first row of electrodes were stirred in acetonitrile face down, the second row face up, and the third row face down with a gap at the bottom (to help the stir bar from bumping the film). The left column uses a small round stir bar, the middle column uses a middle-sized oval stir bar, and lastly, the far right column uses the largest oval stir bar. Clearly, films that are face up in the reaction provide the most robust orientation for the reactions regardless of stir bar size.

Reaction conditions: For each reaction, stock solutions of bromoacetophenone (BrAPN) and triethanolamine (TEOA) in CD_3CN were prepared to minimize error between reactions. For all reactions, a ratio of 1:3:0.002 for BrAPN:TEOA:MO_x-**Ir** was used as depicted in Scheme S2. For a typical nanopowder reaction, 0.0301 mmols of BrAPN from the stock solution was added to the reaction vial with a stir bar, followed by 0.0904 mmols of TEOA, and finally 78 nmols

of nanopowder catalyst added. The mass of catalyst to add was calculated based on the catalyst loadings as determined above. Reaction volume was kept at 1.6 mL. The vials were sealed with a microwave cap sealer. The reaction was then degassed with high purity Ar from a gas cylinder, and the needle holes (from degassing) were sealed with putty. The vial was then placed on a stir plate with the Blue LED 3 cm from the vial. A stir rate of 1000 RPM was set, the fan turned on, and finally, the vial illuminated to initiate catalysis. The light was turned off to stop the reaction. For thin film reactions, the reaction was performed similarly but with 2.6 mL of reaction in order to fully cover the film with solvent in the vial. The nmols of **Ir** on each film were calculated first based on the loadings (see section above) and surface area of the electrode (calculated in ImageJ). Since the thin film catalysts had some variations in loadings depending on the metal oxide used, the absolute quantity of BrAPN and TEOA could vary between the reactions; however the ratio between reagents was kept constant. **Ir** catalyst concentration varied from 10-13 μM in the thin film reactions. Keep in mind that only one film could be added at a time to the reaction, limiting the quantity of catalyst that could be added. This is in contrast to the nanopowders, where one can add more mgs of catalyst to reach the desired nmols. Regardless, the ratios between BrAPN:TEOA:MO_x-Ir were always kept constant as stated above (1:3:0.002) for all reactions-both films and nanopowders.

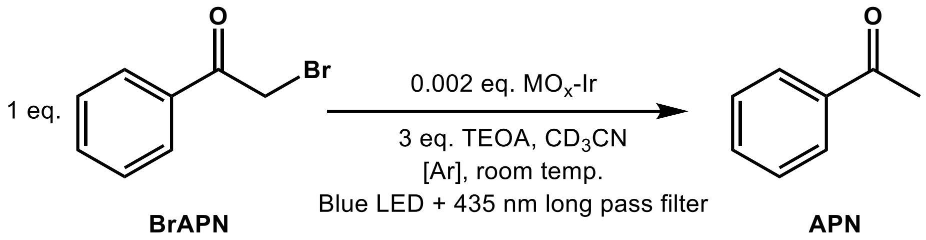

Scheme S2. Reaction conditions for reductive dehalogenation of bromoacetophenone (BrAPN) to acetophenone (APN).

^{1}H NMR quantification: To quantify the reactions, the residual solvent signal was used as an NMR concentration reference.[6] To do this, first a ^{1}H NMR spectrum was collected for a known concentration of APN (0.0188 M) in CD_3CN. The APN singlet at δ 2.56 ppm and the solvent residual pentet at δ1.94 ppm were integrated. The solvent signal was normalized to $\int_{solvent} = 1$, resulting in $\int_{APN} = 0.59$ for a 0.0188 M concentration. This ratio may change slightly from bottle to bottle of deuterated solvent, and was thus, remeasured every time a new bottle was used for the reactions. For each reaction, 0.6 mL of the reaction mixture was added to the NMR tube for analysis. The concentration of APN in the NMR tube was calculated by normalizing the solvent residual pentet to 1, and integrating the APN singlet. Since we knew $\int_{APN} = 0.59$ corresponded to a 0.0188 M APN in the NMR tube, the concentration of APN in the NMR tube could be calculated. Finally, since the exact volume of each reaction was known, the mass of APN produced could be calculated, and finally the reaction yields.

Note that we also double-checked this quantification method using a second quantification method on a test reaction by adding a 3 mM cyclohexene internal standard (δ 5.65 ppm) to the NMR tube and comparing this to an NMR of the same reaction without the cyclohexene internal standard (Figure S9). Nearly identical APN concentrations were found from both the residual solvent signal and internal standard methods, demonstrating the validity of the residual solvent

signal method. We wanted to avoid using an extra internal standard to prevent possible reactivity of the internal standard (especially during reactions tracked over time).

Tracking the reaction: For reactions studied over time, the reactions were setup as described above. The reaction was illuminated for the amount of time required, lamp turned off, and 0.6 mL of the reaction removed for ^{1}H NMR. After, the reaction mixture in the NMR tube was readded to the reaction mixture, a new cap was placed on the vial, and the reaction degassed with Ar. This process was repeated for each data point. Representative NMRs of this are shown in Figure S8.

Ir homogeneous reaction. A reaction was also run with **Ir** in solution as a homogeneous catalyst. The reaction was run identical to the nanopowder catalyst reactions with a ratio of 1:3:0.002 for BrAPN:TEOA:**Ir**. This reaction was also followed over time to compare it to the heterogenized catalysts (Table S3, Figure S5).

Table S3. Reaction yields for reductive dehalogenation of bromoacetophenone to acetophenone with MO_x-**Ir** nanopowders over time and the **Ir** homogeneous control reaction.

**Time (min)**	**% Yield APN for ITO-Ir**	**% Yield APN for Al_2O_3-Ir**	**% Yield APN for ZrO_2-Ir**	**% Yield APN for Ir homogeneous**
0	0	0	0	0
1	2	32	18	18
2	6	62	41	28
3	11	85	60	34
6	18	97	83	52
15	25	99	93	85
30	39	100	100	96
60	91	100	100	100
120	100	100	100	100

Table S4. Reaction yields for reductive dehalogenation of bromoacetophenone to acetophenone with MO_x-**Ir** films.

**Time (min)**	**% Yield APN for ITO-Ir**	**% Yield APN for Al_2O_3-Ir**	**% Yield APN for ZrO_2-Ir**
0	0	0	0
15	8	12	22
30	32	23	36
60	71	59	65
120	98	82	65
240	100	94	87

Photoredox Catalysis Plots

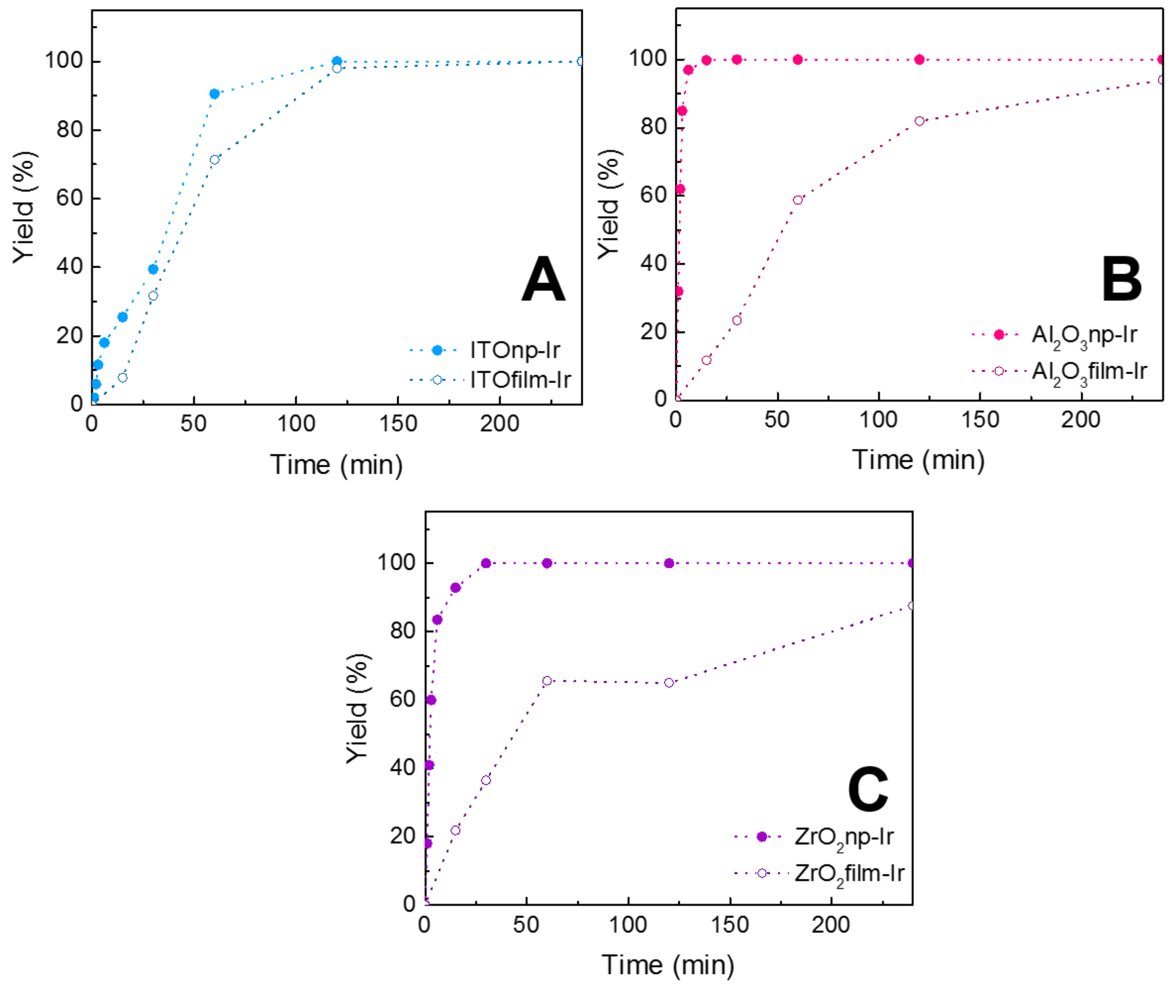

Figure S4. Reaction yields overtime comparing thin films (open circles) and nanopowder catalysts (filled circles) for (A) ITO-**Ir** (blue) (B) Al_2O_3-**Ir** (pink), (C) ZrO_2-**Ir** (purple).

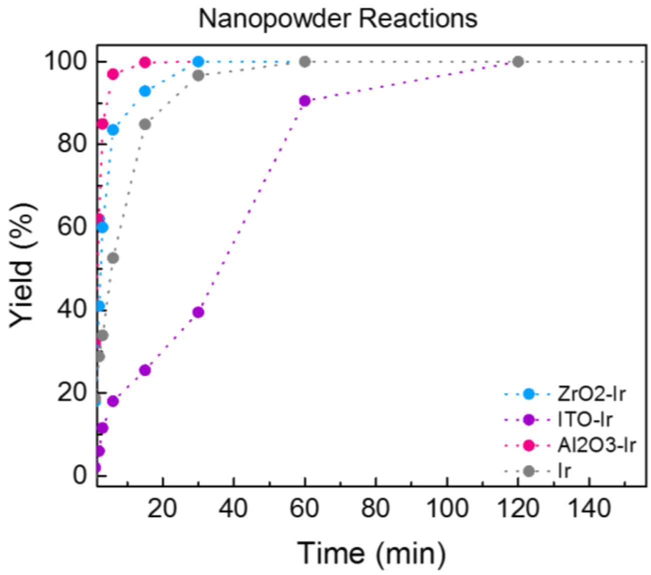

Figure S5. Reaction yield over time during photoredox catalysis of nanopowders catalysts ITO-**Ir** (blue), Al_2O_3-**Ir** (pink), and ZrO_2-**Ir** (purple). Homogeneous reaction with **Ir** in solution is shown in grey.

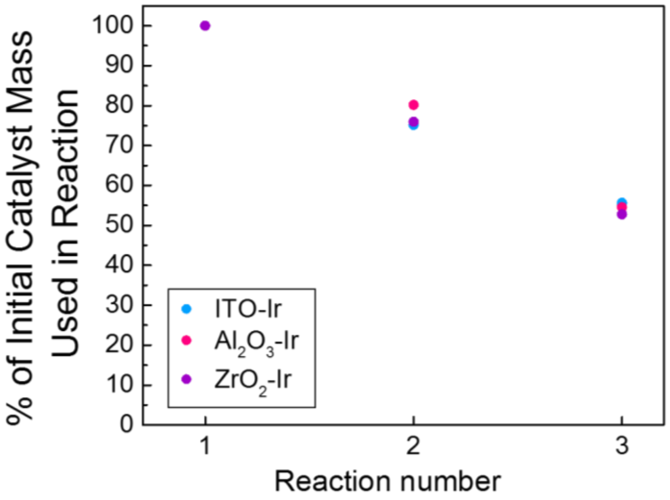

Figure S6. Percent of the initial nanopowder catalyst mass (mg) used at the start of each reaction used in the reusability experiments for ITO-**Ir** (blue), Al_2O_3-**Ir** (pink), ZrO_2-**Ir** (purple). A loss in the mass (mgs) of catalyst was observed after each catalytic test and was likely due to the nanopowders sticking to sides of flask and the centrifuge tube.

Additional Spectra

^{1}H NMR Spectra

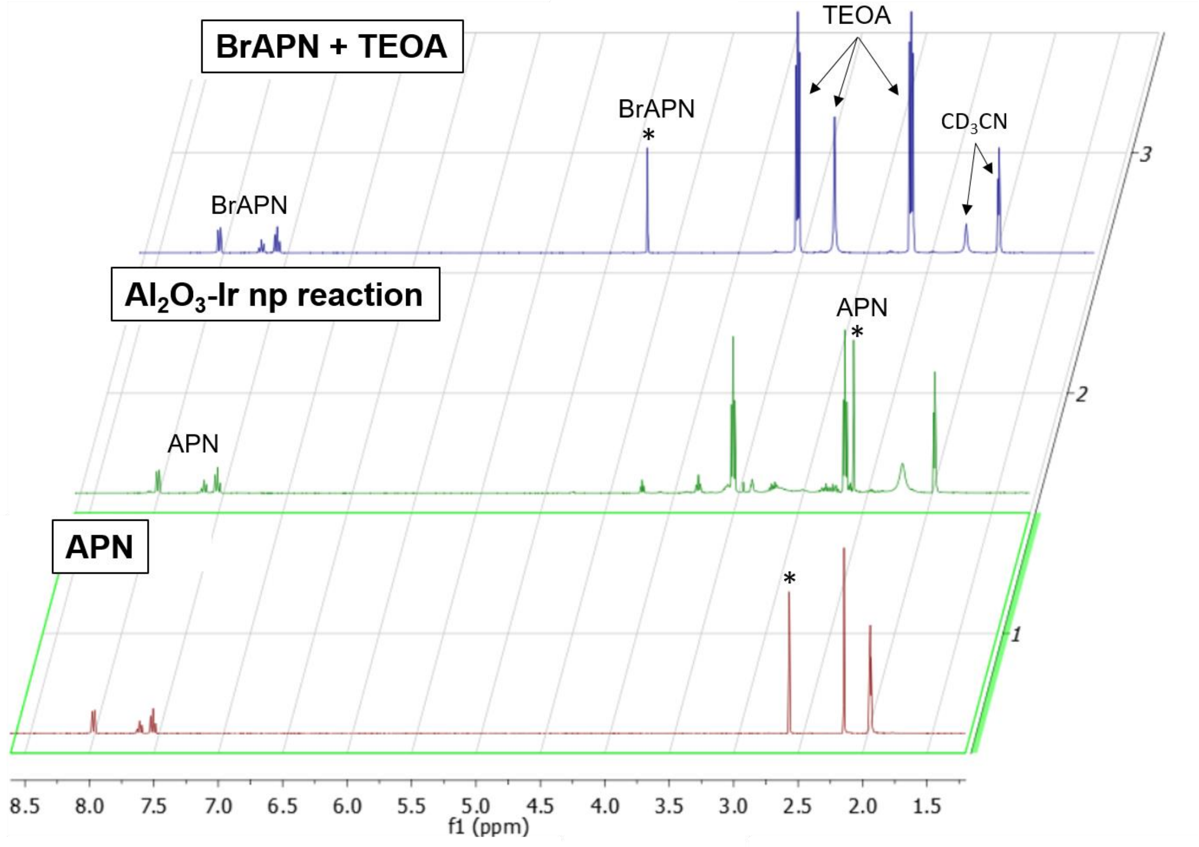

Figure S7. Representative ^{1}H NMR spectra of (top) starting reaction, (middle) post reaction with Al_2O_3-**Ir** nanopowder catalyst, and (bottom) acetophenone (APN) in CD_3CN. BrAPN, TEOA, APN, and CD_3CN peak assignments are labeled for clarity. The disappearance of the BrAPN singlet is seen after photoredox catalysis (middle) and the appearance of the product APN singlet (middle).

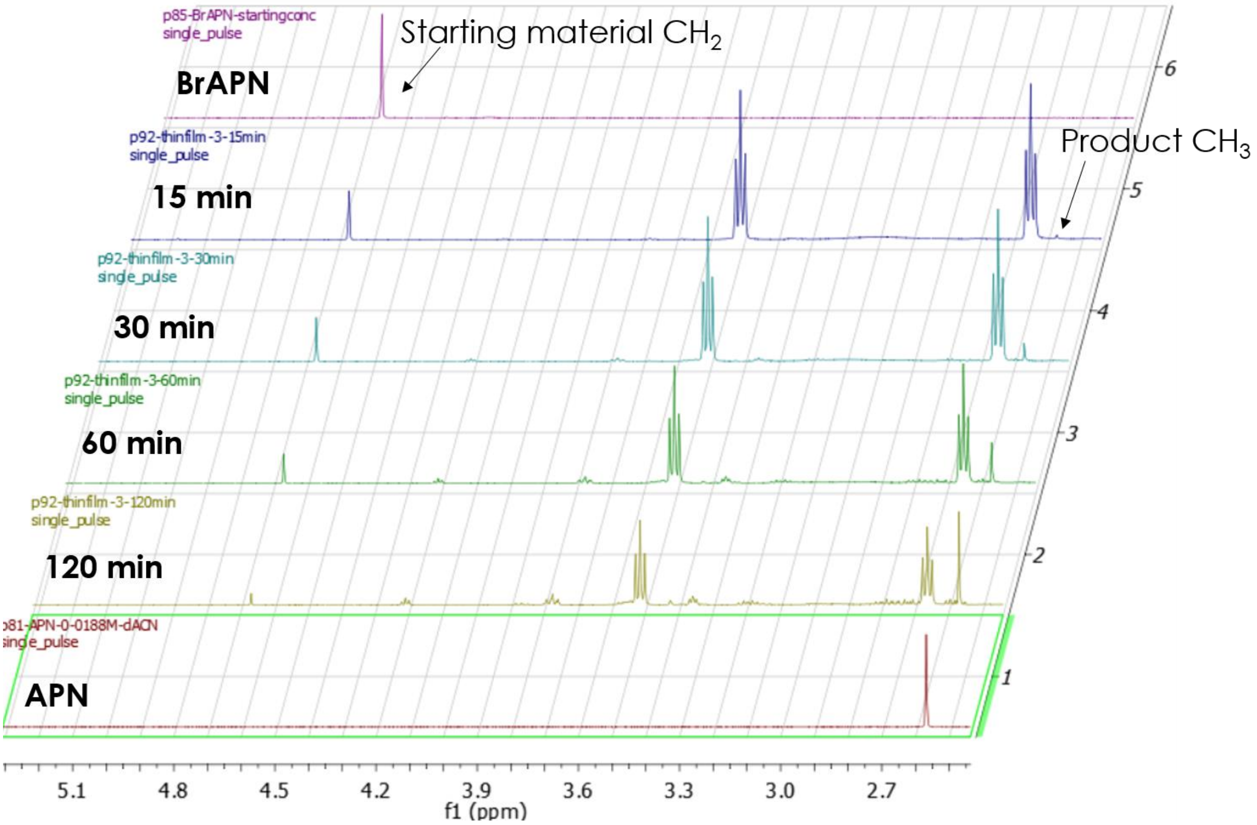

Figure S8. Representative example of ^{1}H NMR over time using Al_2O_3-**Ir** thin films. Starting material peak at δ 4.68 ppm decreases with time, while the product peak at δ 2.56 ppm grows in with time. Top ^{1}H NMR is of BrAPN and bottom is of APN in CD_3CN.

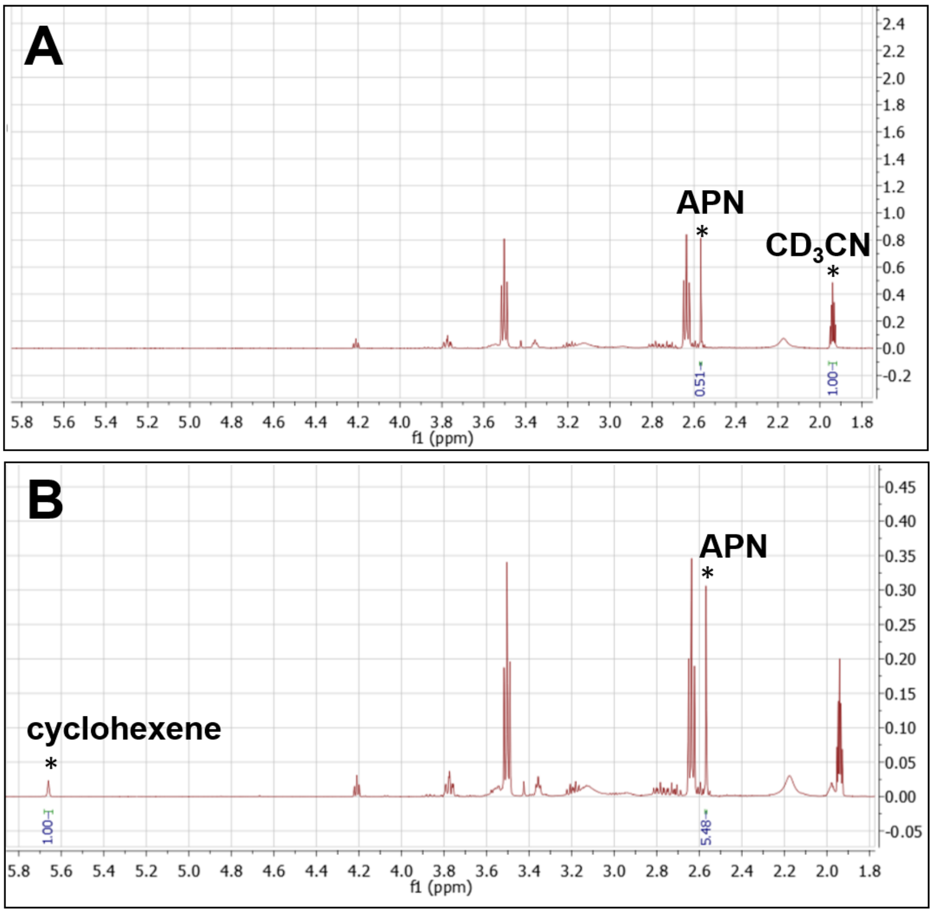

Figure S9. ^{1}H NMR of a photoredox reaction using Al_2O_3-**Ir** nanopowder catalysts after fifteen minutes of illumination using (A) the residual solvent signal to quantify the APN yield and (B) using a 3 mM cyclohexene internal standard to quantify the APN yield. Integrations of APN, CD_3CN and cyclohexene are shown in each panel and were used in the quantifications. Using the residual solvent signal method (as described above), we obtain a 0.0163 M concentration of APN in the NMR tube, and the cyclohexene internal standard method gives a concentration of 0.0164 M.

UV-Vis spectroscopy

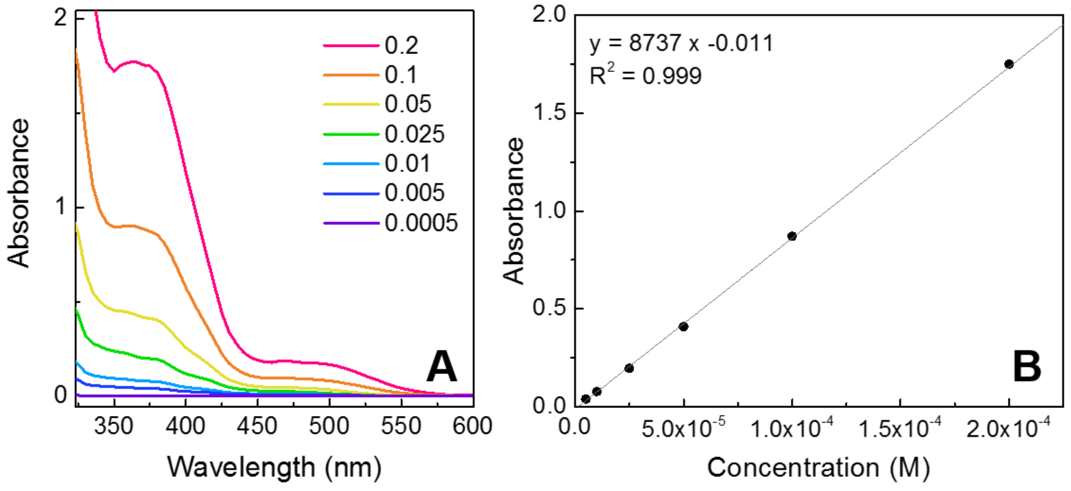

Figure S10. (A) UV-Vis spectra of **Ir** at ranging concentrations (depicted in mM in the legend from 0.2 mM to 0.0005 mM). (B) Beer-Lambert plot of absorbance versus concentration at 370 nm; this results in an extinction coefficient for **Ir** of 8737 M^{-1} cm^{-1} at this wavelength.

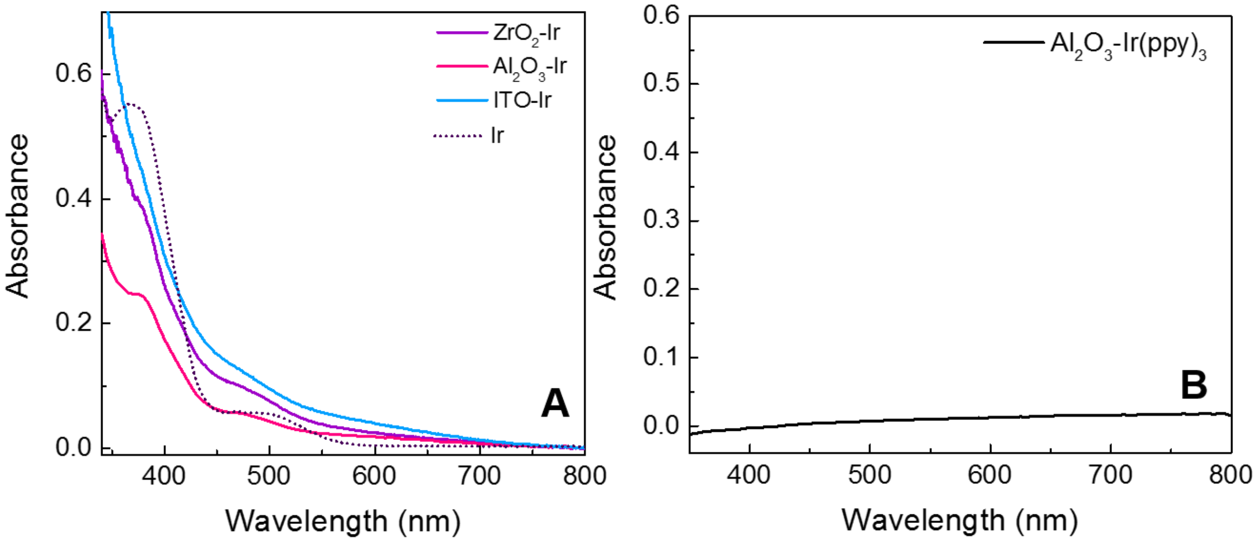

Figure S11. (A) Example of UV-Vis spectra of thin films (MOx background subtracted); ZrO_2-**Ir** (purple), Al_2O_3-**Ir** (pink), ITO-**Ir** (blue), and **Ir** solution spectrum in acetonitrile (black dotted). (B) UV-Vis spectra of a thin film (MOx background subtracted) of Al_2O_3 film soaked in the presence of $Ir(ppy)_3$ (Tris[2-phenylpyridinato-C2,N]iridium(III)), which has *no surface anchor*, in methanol. Film was rinsed with methanol, and the spectrum collected. No $Ir(ppy)_3$ was detected on the electrode suggesting the carboxylic acid anchor in **Ir** is needed for covalent attachment to the metal oxide surface, which is a well-established methodology.[7]

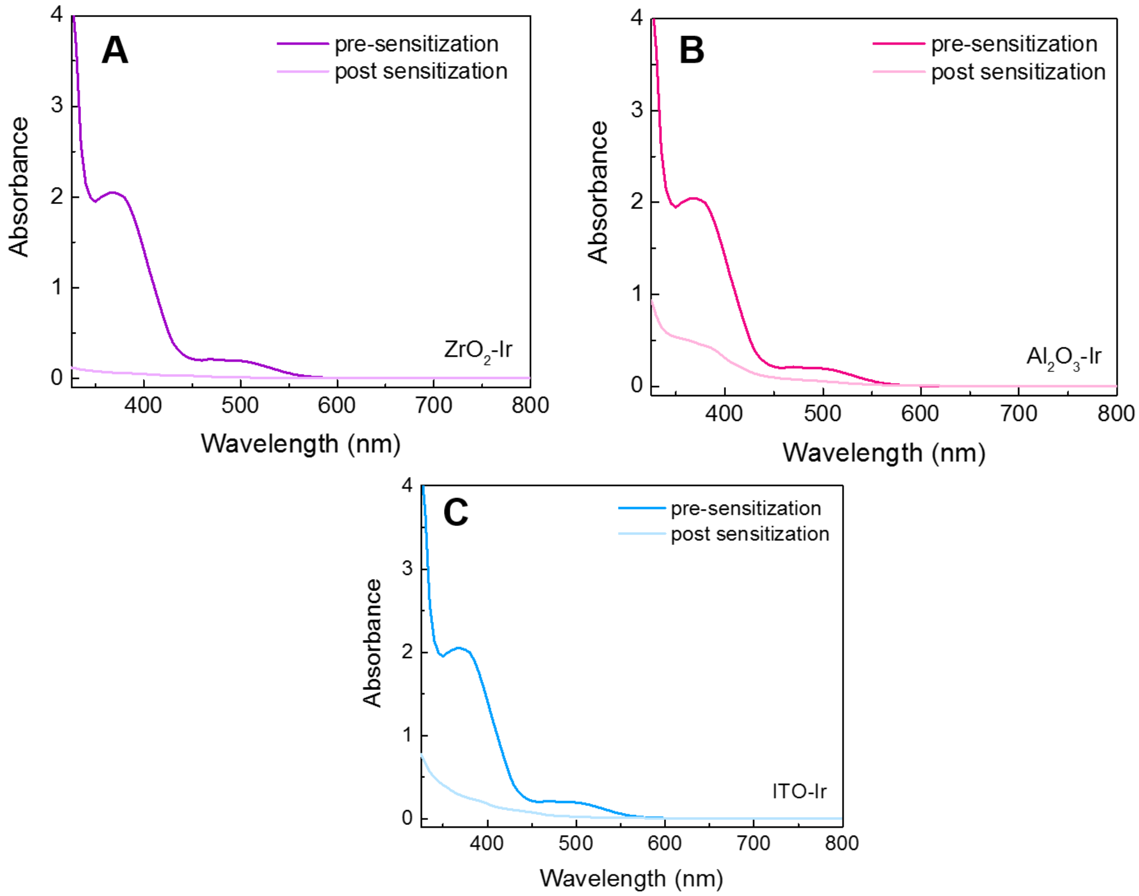

Figures S12. UV-Vis of **Ir** sensitization solutions before (darker color) and after (lighter color) catalyst immobilization (depletion method) for (A) ZrO_2-**Ir** (purple), (B) Al_2O_3-**Ir** (pink), (C) ITO-**Ir** (blue). A decrease in absorption post sensitization suggests that **Ir** has bound to the metal oxide surfaces.

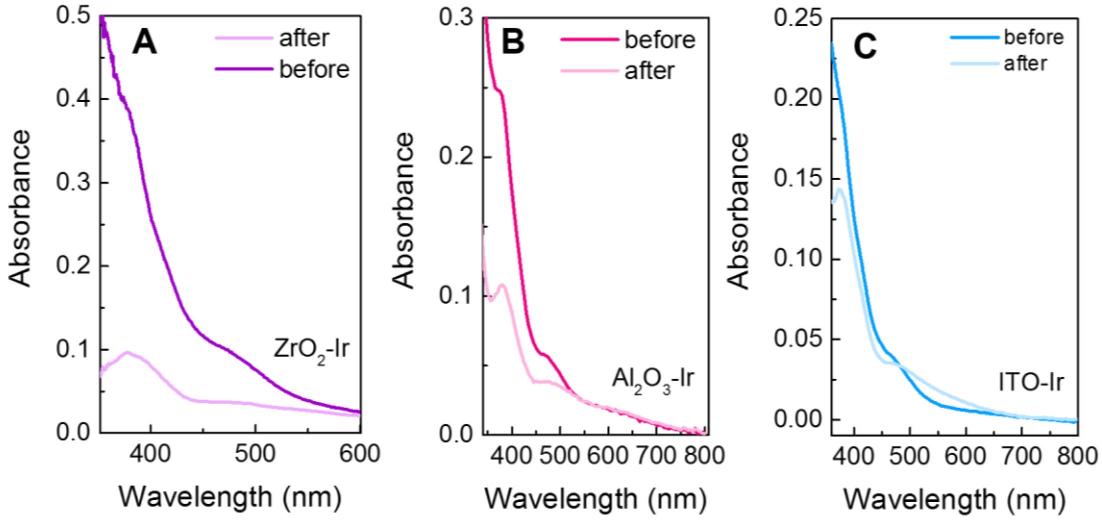

Figure S13. UV-Vis spectra of thin films before (darker color) and after (lighter color) four hours of photoredox catalysis for (A) ZrO_2-**Ir** (purple), (B) Al_2O_3-**Ir** (pink), (C) ITO-**Ir** (blue).

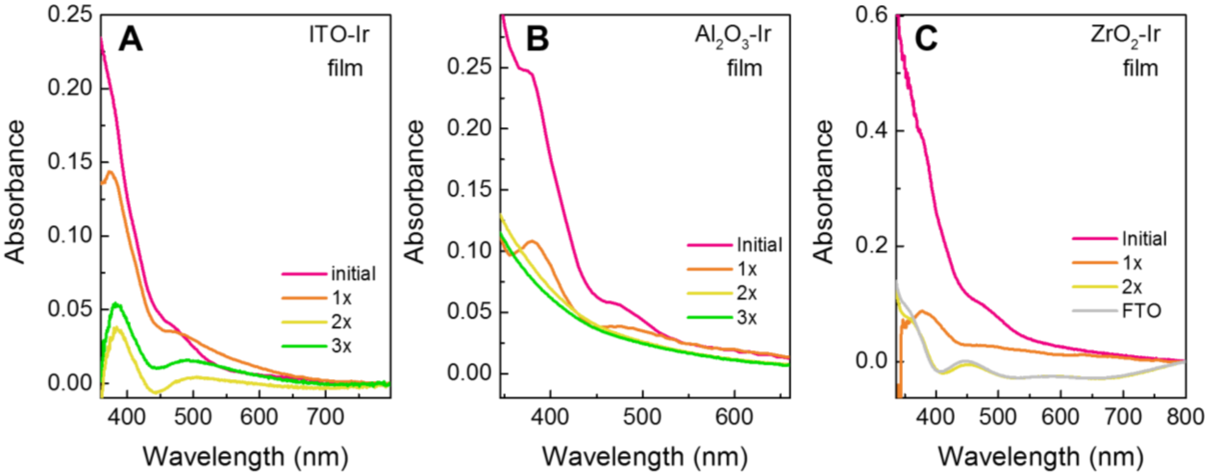

Figure S14. UV-Vis spectra of thin films post reusability tests for (A) ITO-**Ir** (B) Al_2O_3-**Ir** and (C) ZrO_2-**Ir** thin films. Initial UV-Vis spectra of MOx-**Ir** (pink), post first reaction (orange), post second reaction (yellow), and post third reaction (green). ZrO_2 film loss occurs after each reaction, and as a result by the end of the second reaction, only peaks from FTO (grey) are able to be captured by UV-Vis (panel C).

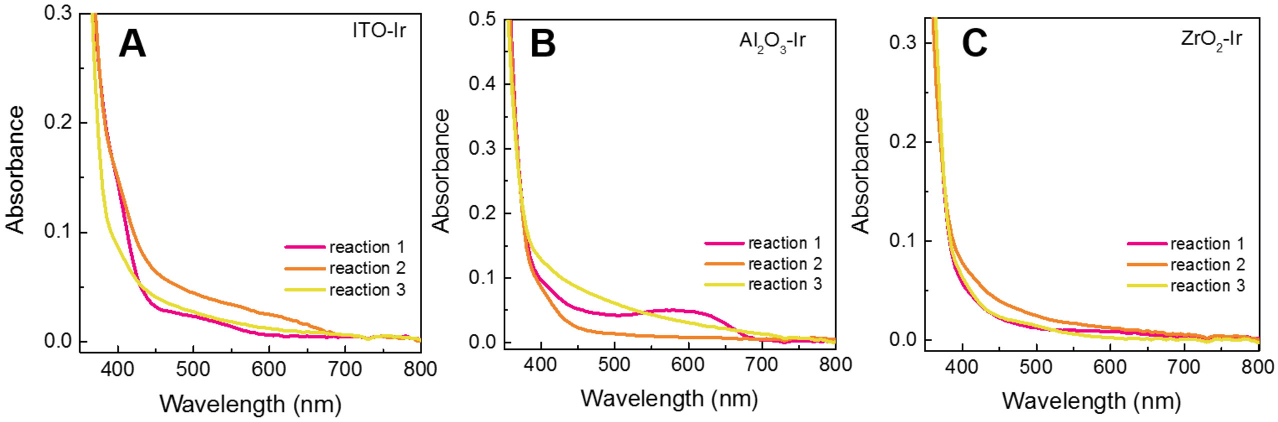

Figure S15. UV-Vis spectra of reaction mixtures post photoredox catalysis after three catalyst reactions for nanopowders (A) ITO-**Ir** (B) Al_2O_3-**Ir** (C) ZrO_2-**Ir**; first reaction mixture (pink), second reaction mixture (orange), and third mixture reaction (yellow).

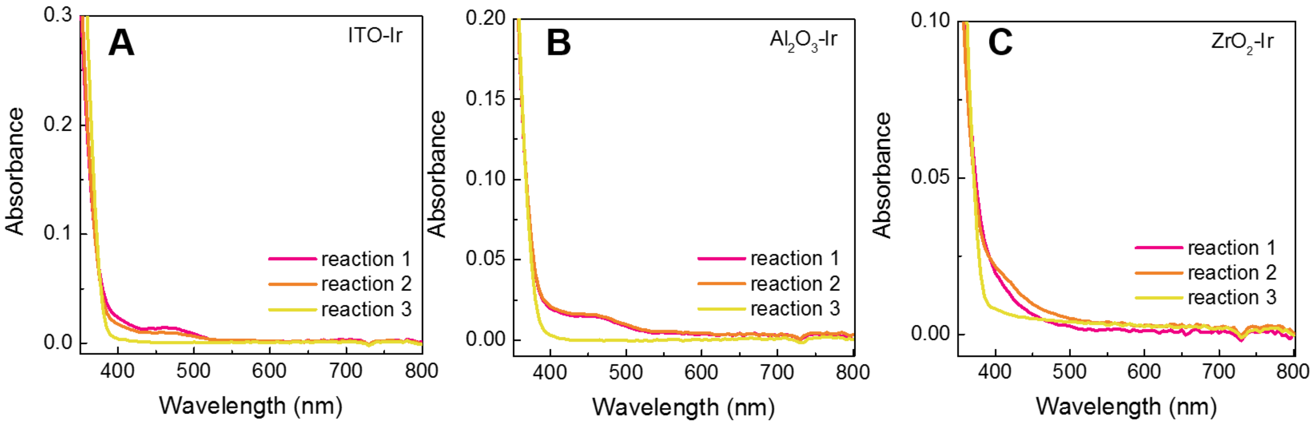

Figure S16. UV-Vis spectra of reaction mixtures post photoredox catalysis after three catalyst reactions for films (A) ITO-**Ir** (B) Al_2O_3-**Ir** (C) ZrO_2-**Ir**; first reaction mixture (pink), second reaction mixture (orange), and third mixture reaction (yellow).

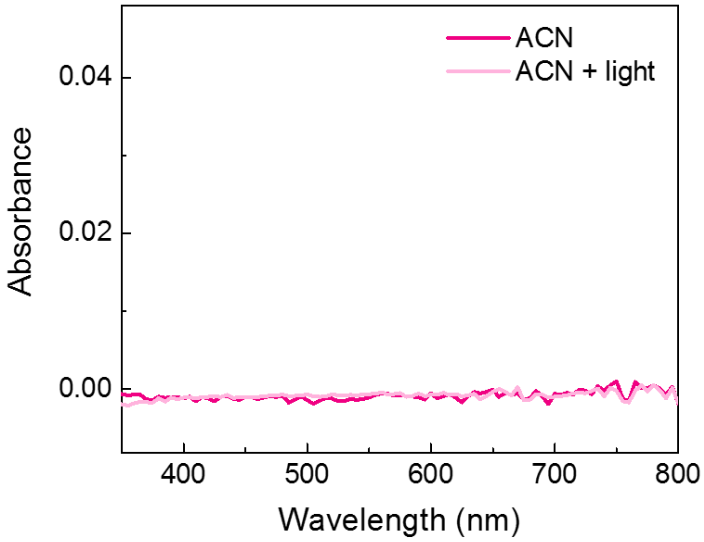

Figure S17. UV-Vis of acetonitrile post soaking electrodes 24 hours (dark pink) and UV-vis of acetonitrile post illumination with a white LED of metal oxides for 24 hours in acetonitrile (light pink).

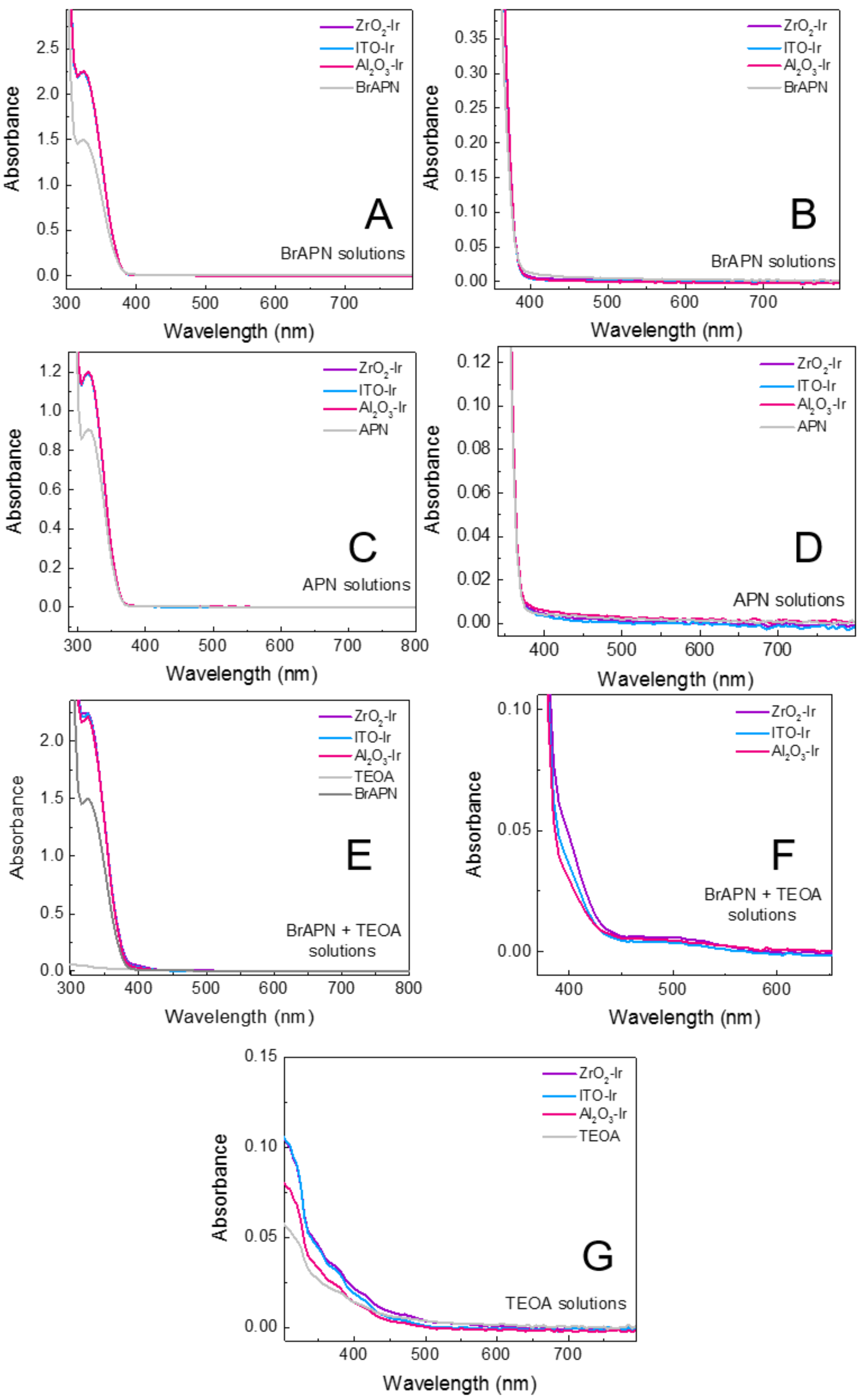

Figure S18. UV-Vis of acetonitrile solutions of BrAPN (A,B), APN (C,D) BrAPN+TEOA (E,F) and TEOA (G) and after soaking films in them for two hours. Concentrations were

identical to those used in photoredox tests. Panels B, D, and F are *zoomed in* plots of A, C, and E, respectively. Reference spectra of pure TEOA, BrAPN, or APN dissolved in acetonitrile are also depicted in each plot in grey.

ATR-FTIR spectroscopy

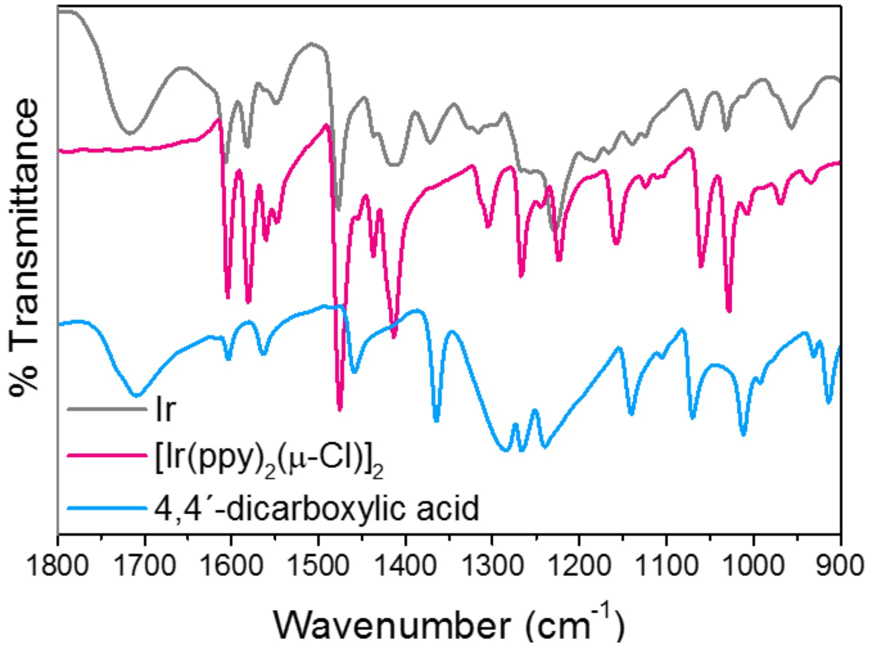

Figure S19. ATR-FTIR spectra of powders of **Ir** (grey), ([Ir(ppy)$_2$(μ-Cl)]$_2$) (pink) and 4,4´-dicarboxylic acid (blue). C=O stretching bands are observed in **Ir** and 4,4´-dicarboxylic acid at 1709 cm^{-1}. C=C stretches are observed in **Ir** at 1607 cm^{-1} and C=N stretches at 1583 cm^{-1}.

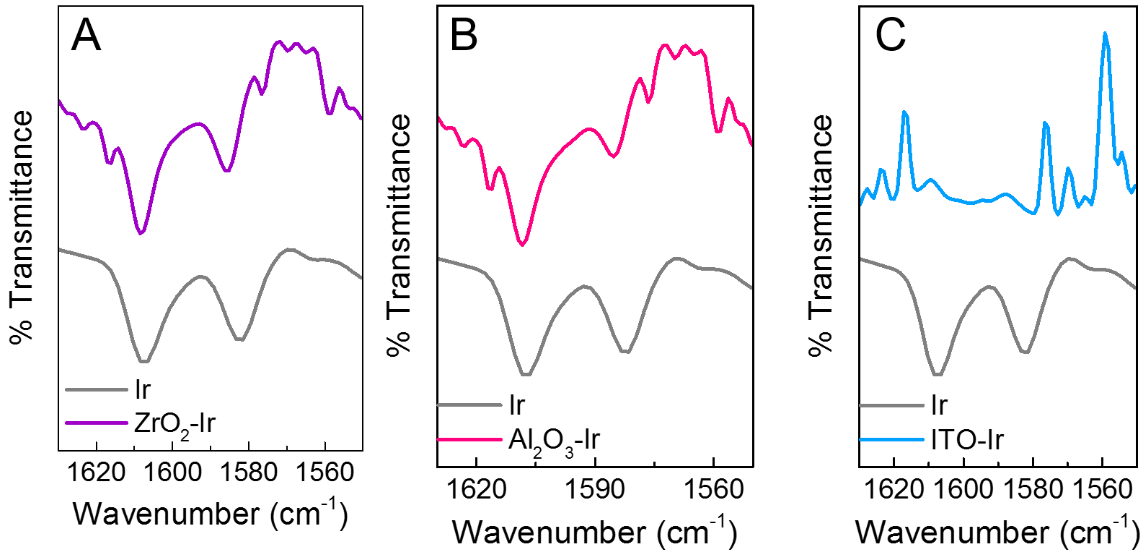

Figure S20. ATR-FTIR spectra of thin films for (A) ZrO$_2$-**Ir** (purple) (B) Al$_2$O$_3$-**Ir** (pink) (C) ITO-**Ir** (blue). **Ir** powder prior to surface binding is shown in grey in each panel. C=C stretches are observed in **Ir** and MO$_x$-**Ir** at 1607 cm^{-1} and C=N stretches at 1583 cm^{-1}.

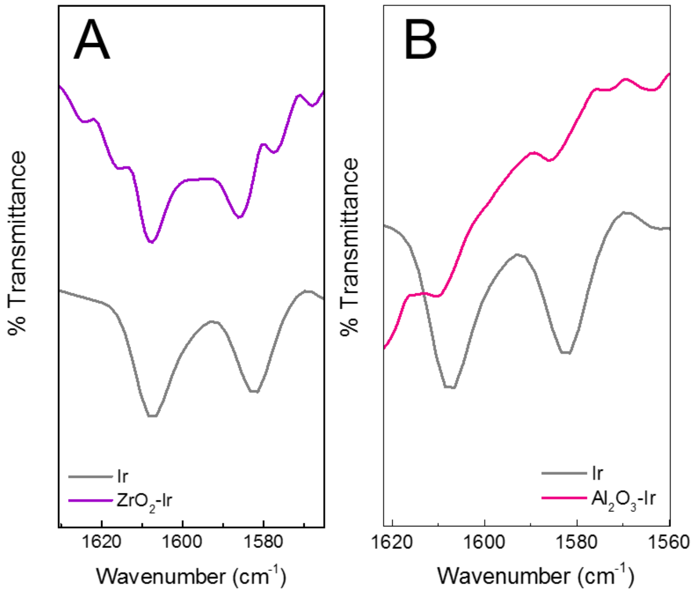

Figure S21. ATR-FTIR spectra of nanopowders for (A) ZrO_2-**Ir** (purple) and (B) Al_2O_3-**Ir** (pink). **Ir** powder is shown in grey in each panel. C=C stretches are observed in **Ir** and MO_x-**Ir** at 1607 cm^{-1} and C=N stretches at 1583 cm^{-1}. The peaks in Al_2O_3-**Ir** are less intense likely due to the lower loading of **Ir** on Al_2O_3 versus ZrO_2. ATR-FTIR of ITO-**Ir** was unsuccessful likely due to surface plasmon resonance effects of ITO or due to an inhomogeneous loading of **Ir** on ITO making it difficult to detect peaks.

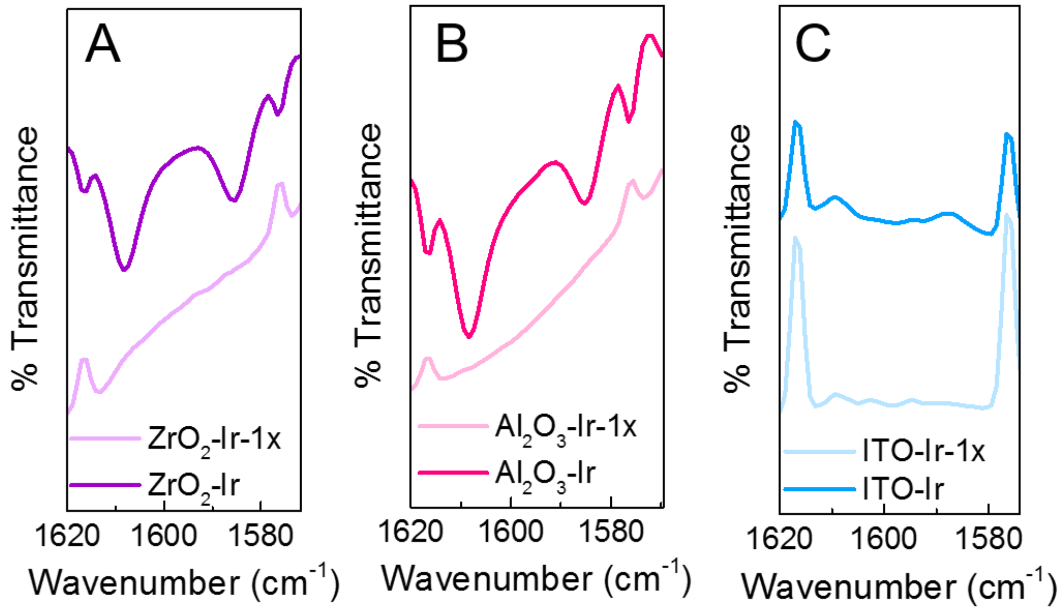

Figure S22. ATR-FTIR spectra of thin films post photoredox catalysis (labeled "1x" for one use) for (A) ZrO_2-**Ir** (purple) and (B) Al_2O_3-**Ir** (pink) and (C) ITO-**Ir**. C=C stretches are observed in the initial MO_x-**Ir** at 1607 cm^{-1} and C=N stretches at 1583 cm^{-1}; these peaks difficult to detect after one catalytic test for the ZrO_2 and Al_2O_3 films, likely due to loss of some **Ir** from the surface.

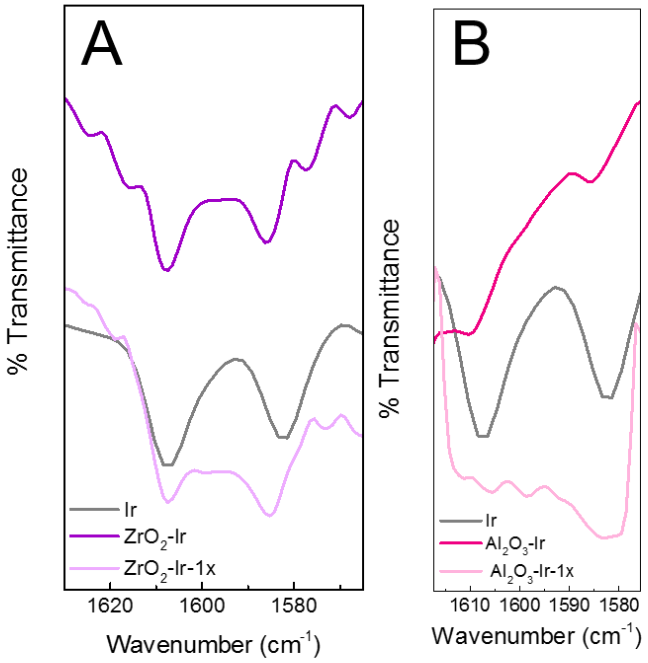

Figure S23. ATR-FTIR spectra of nanopowders post photoredox catalysis (labeled “1x” for one use) for (A) ZrO_2-**Ir** (purple) and (B) Al_2O_3-**Ir** (pink); **Ir** powder is shown in grey in each panel. C=C stretches are observed in **Ir** and initial MO_x-**Ir** at 1607 cm^{-1} and C=N stretches at 1583 cm^{-1}; these peaks are retained after one catalytic test for ZrO_2-**Ir** nanopowder. The peaks in Al_2O_3-**Ir** are less intense likely due to the lower loading of **Ir** on Al_2O_3 versus ZrO_2; peaks are also retained after photoredox tests for Al_2O_3-**Ir**, but more peaks also appear, suggesting some changes on the surface.

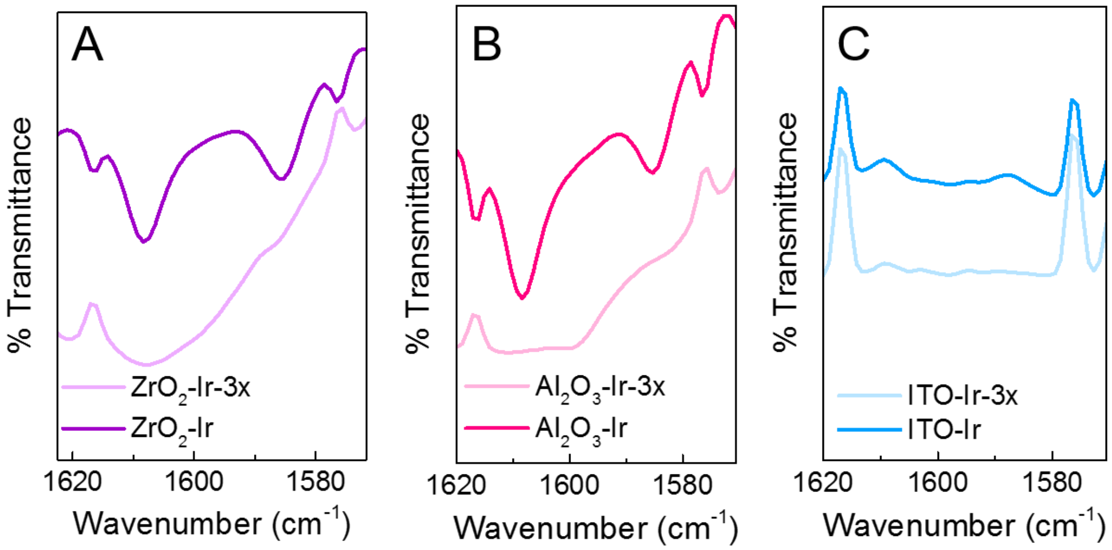

Figure S24. ATR-FTIR spectra of thin films post photoredox catalysis three times (labeled “3x” for three uses) from reusability tests for (A) ZrO_2-**Ir** (purple), (B) Al_2O_3-**Ir** (pink) and (C) ITO-**Ir**. C=C stretches are observed in the initial MO_x-**Ir** at 1607 cm^{-1} and C=N stretches at 1583 cm^{-1}; these peaks difficult to detect after three catalytic tests for the ZrO_2 and Al_2O_3 films, likely due to loss of some **Ir** from the surface.

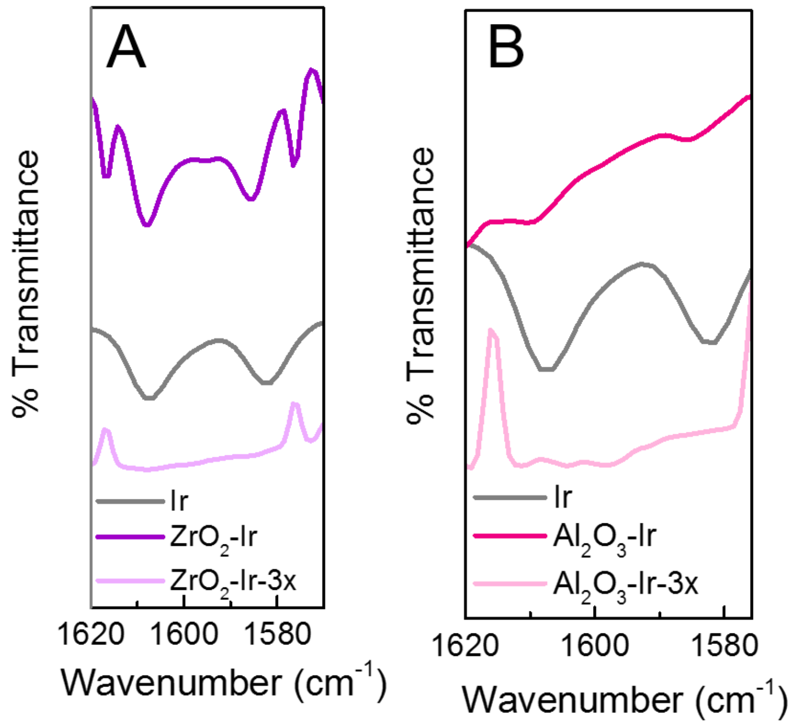

Figure S25. ATR-FTIR spectra of nanopowders post reusability tests. ATR-FTIR spectra of thin films post photoredox catalysis three times (labeled "3x" for three uses) from reusability tests for (A) ZrO_2-**Ir** (purple) and (B) Al_2O_3-**Ir** (pink). C=C stretches are observed in the initial MO_x-**Ir** at 1607 cm^{-1} and C=N stretches at 1583 cm^{-1}; these peaks difficult to detect after three catalytic tests for the ZrO_2 and Al_2O_3 films, likely due to loss of some **Ir** from the surface.

X-ray photoelectron spectroscopy (XPS)

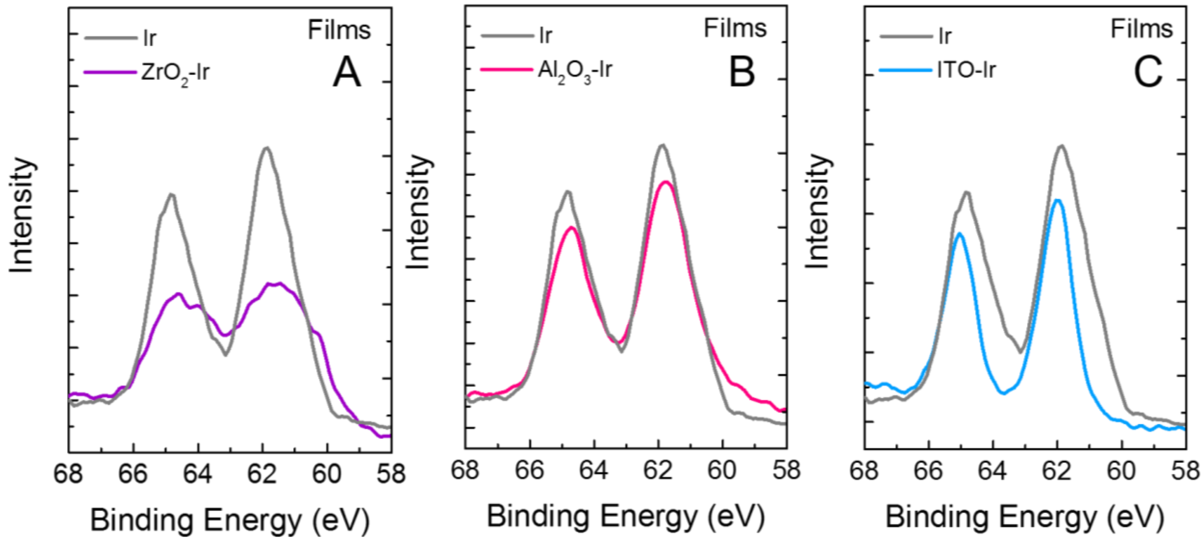

Figure S26. XPS spectra of the Ir 4f region for thin film catalyst of (A) ZrO_2-**Ir** (purple), (B) Al_2O_3-**Ir** (pink) and (C) ITO-**Ir**. **Ir** powder is shown in grey on all traces.

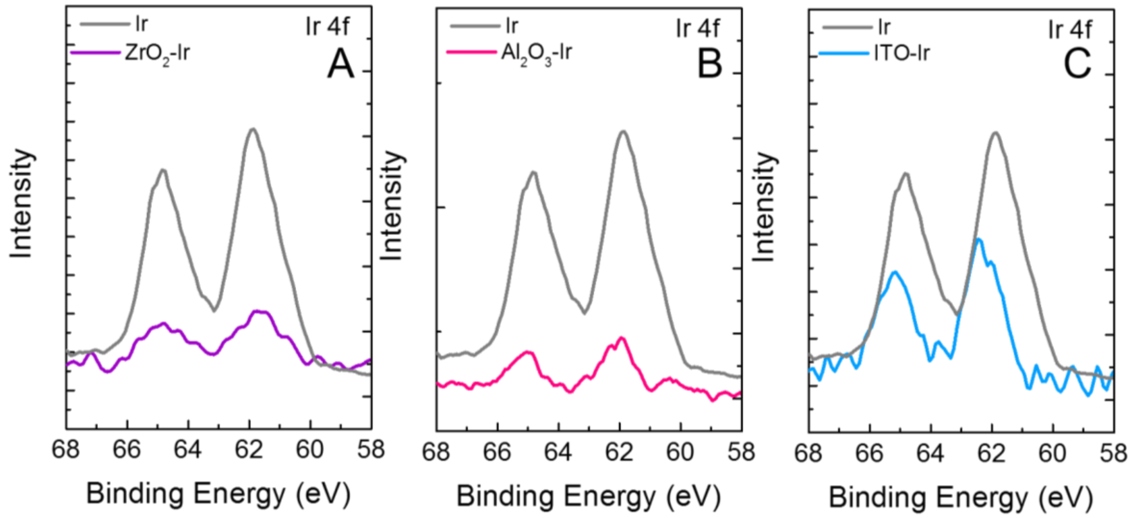

Figure S27. XPS spectra of the Ir 4f region for nanopowder catalysts of (A) ZrO_2-**Ir** (purple), (B) Al_2O_3-**Ir** (pink) and (C) ITO-**Ir** (blue). **Ir** powder is shown in grey on all traces.

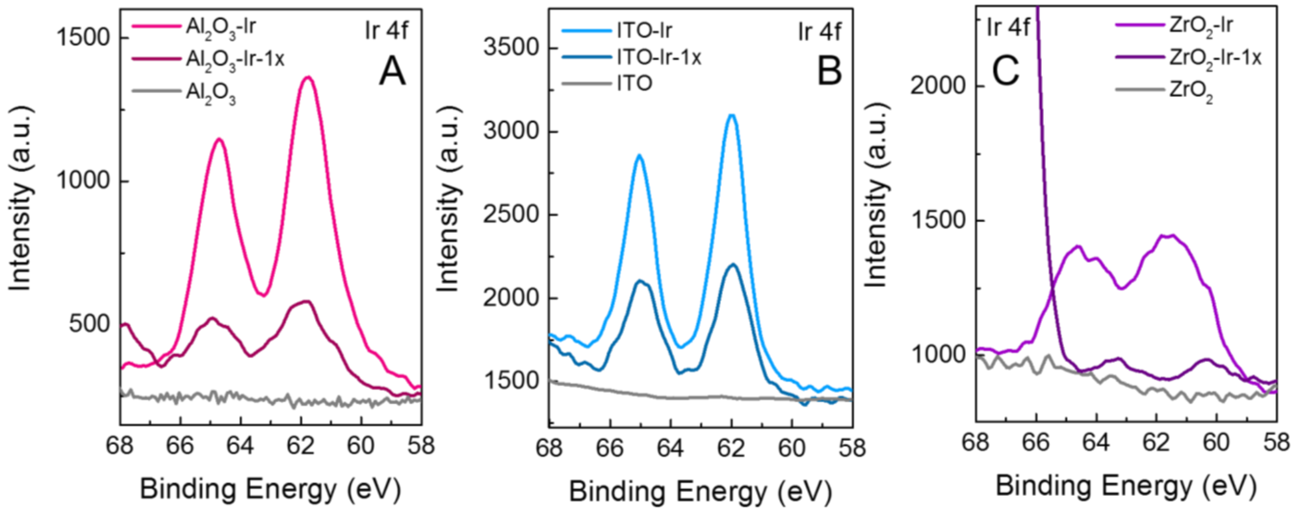

Figure S28. XPS spectra of the Ir 4f region for thin film catalysts of (A) ZrO_2-**Ir** (purple), (B) Al_2O_3-**Ir** (pink) and (C) ITO-**Ir** (blue) before (lighter color) and after one catalytic test (darker color). Baseline metal oxide spectra are shown in grey.

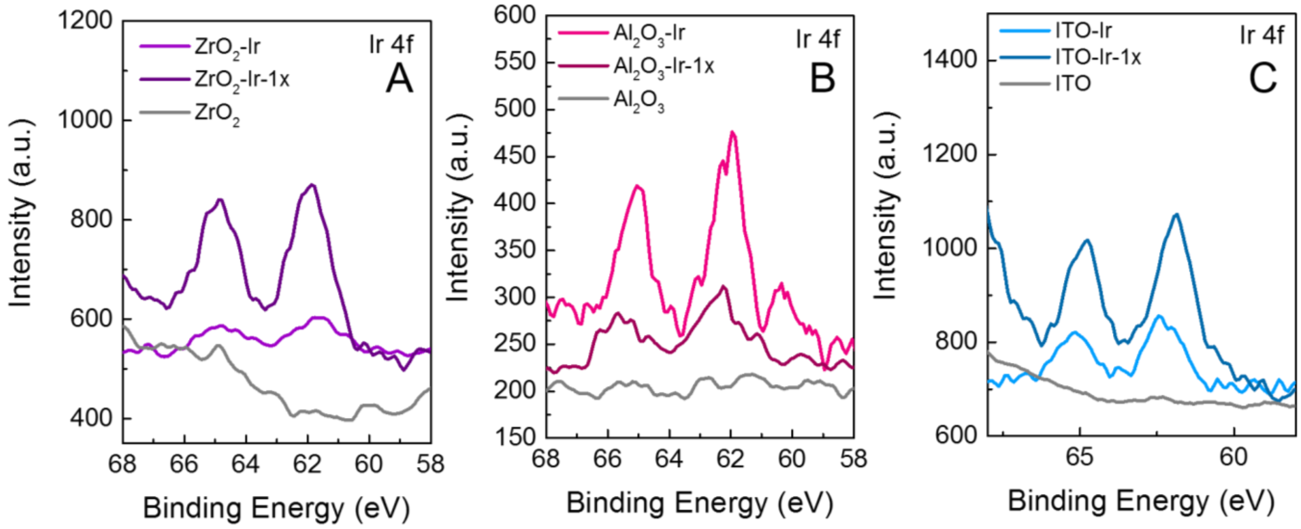

Figure S29. XPS spectra of the Ir 4f region for nanopowder catalysts of (A) ZrO_2-**Ir** (purple), (B) Al_2O_3-**Ir** (pink) and (C) ITO-**Ir** (blue) before (lighter color) and after one catalytic test (darker color). Baseline metal oxide spectra are shown in grey.

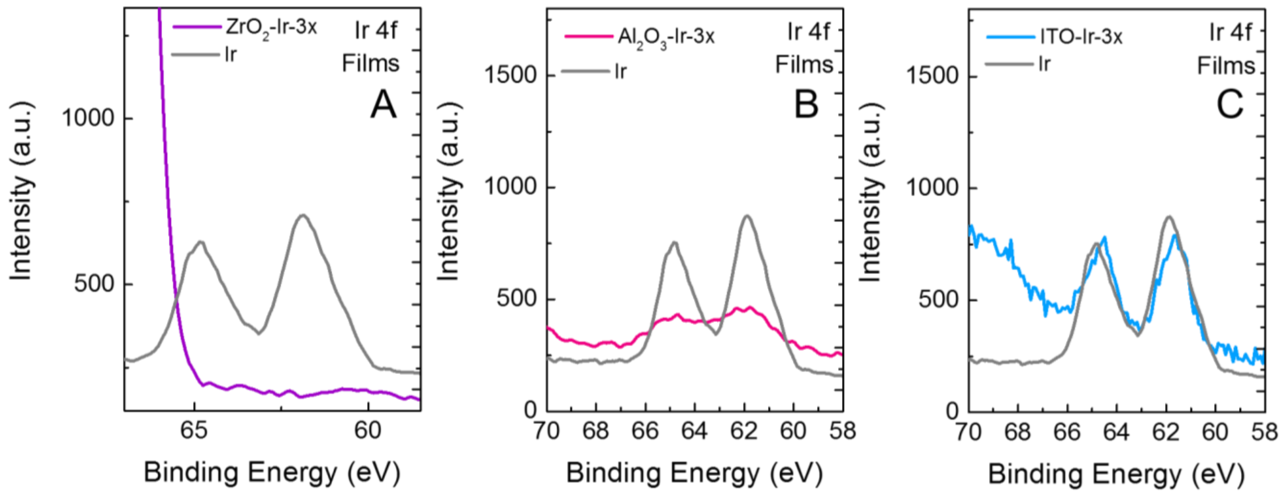

Figure S30. XPS spectra of the Ir 4f region for thin film catalysts post reusability tests for (A) ZrO_2-**Ir** (purple), (B) Al_2O_3-**Ir** (pink) and (C) ITO-**Ir** (blue) after three uses. **Ir** powder blank is shown in grey in each figure.

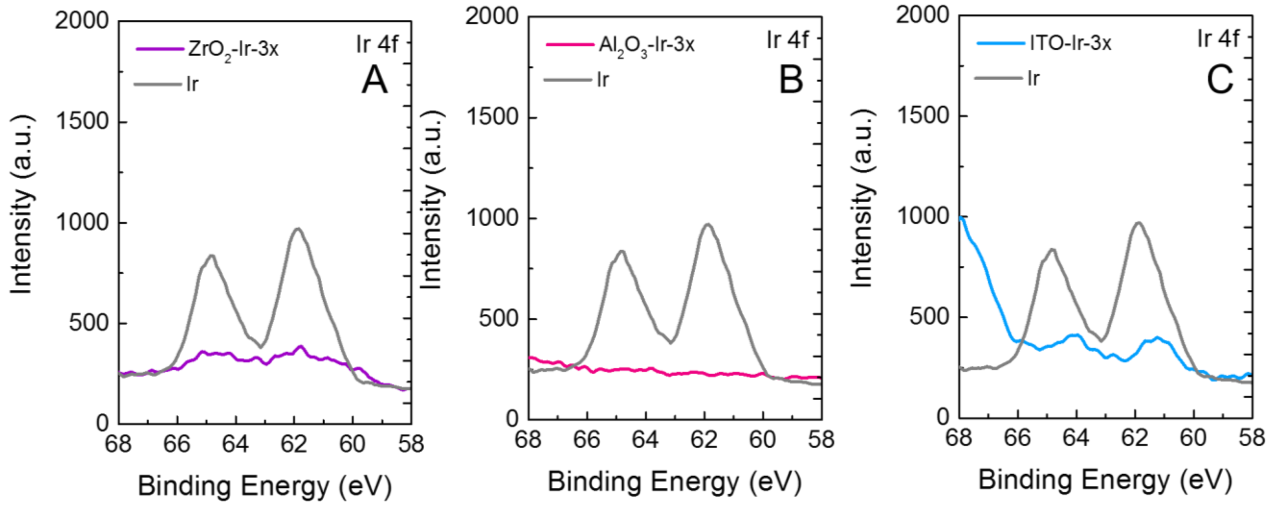

Figure S31. XPS spectra of the Ir 4f region for nanopowder catalysts post reusability tests for (A) ZrO_2-**Ir** (purple), (B) Al_2O_3-**Ir** (pink) and (C) ITO-**Ir** (blue) after three uses. **Ir** powder blank is shown in grey in each figure.

Cyclic voltammetry

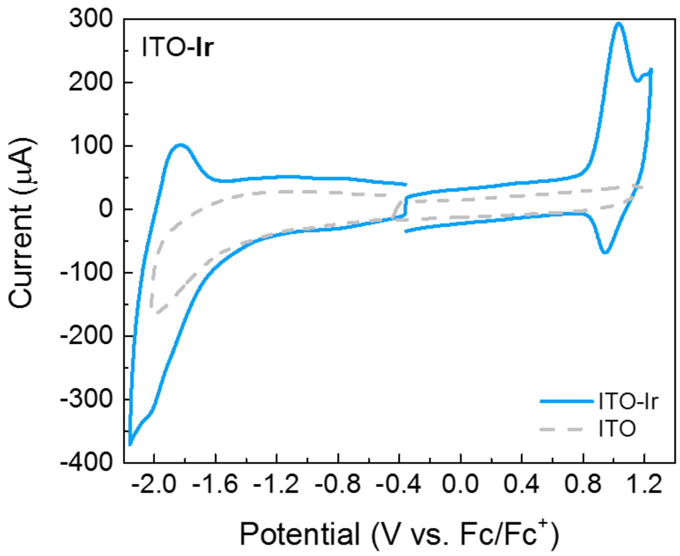

Figure S32. Cyclic voltammogram of ITO-**Ir** (blue) and ITO (grey) in a 0.1 M $TBAPF_6$ electrolyte in acetonitrile with a scan rate of 20 mV/s under Ar. Ferrocene was used as an internal standard.

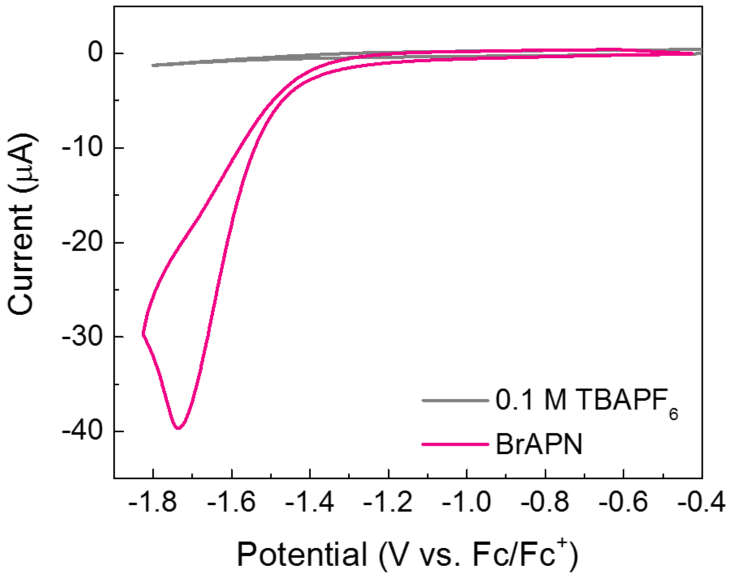

Figure S33. Cyclic voltammogram of 2 mM BrAPN (pink) in a 0.1 M $TBAPF_6$ electrolyte in acetonitrile with a scan rate of 20 mV/s under Ar. The blank electrolyte CV is depicted in grey. Ferrocene was used as an internal standard.

Time-correlated single photon counting (TCSPC)

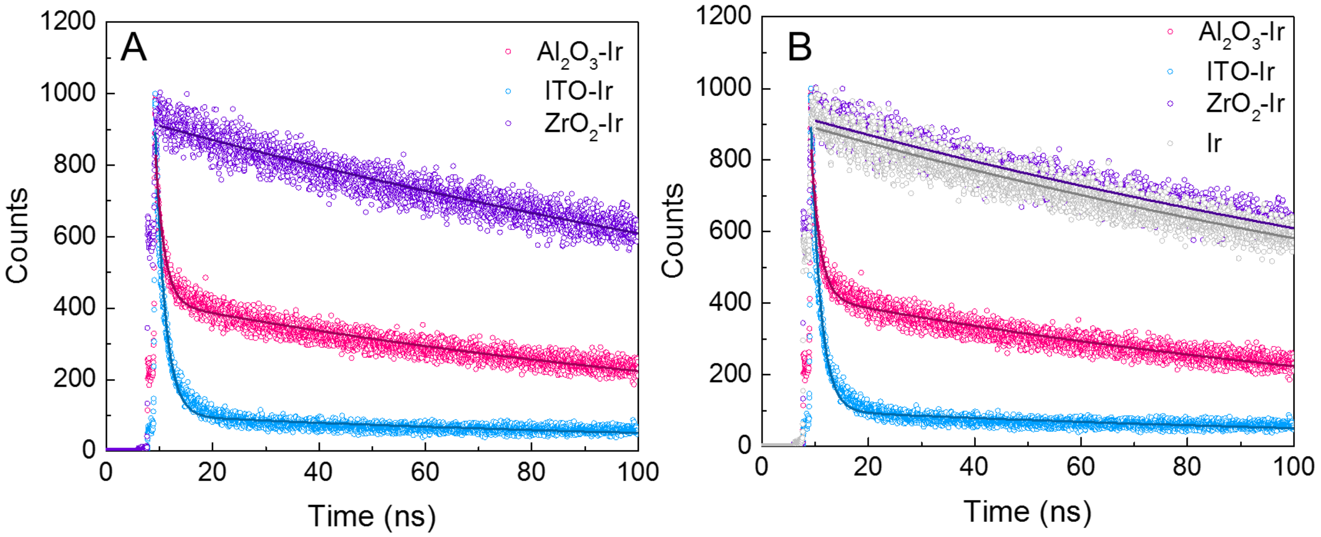

Figure S34. (A) Photoluminescence decay of thin film samples of ITO-**Ir** (blue), Al_2O_3-**Ir** (pink), and ZrO_2-**Ir** (purple) after excitation at 470 nm. (B) Fluorescence decay of ITO-**Ir** (blue) , Al_2O_3-**Ir** (pink), and ZrO_2-**Ir** (purple), with comparison to **Ir** (grey) in acetonitrile after excitation at 470 nm; a 550 nm long pass filter was used for detection.. Data was fit to single or biexponential functions (solid lines); details can be found in Table S5.

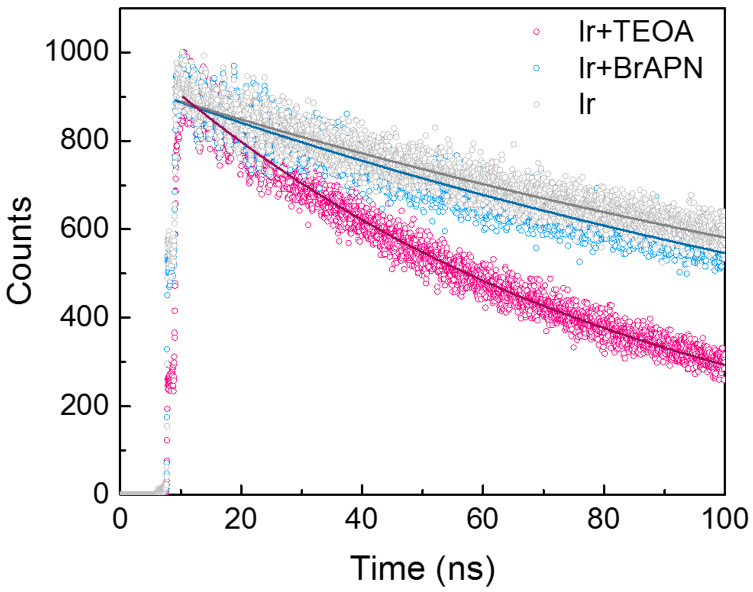

Figure S35. Photoluminescence decay of solution phase samples of **Ir** (grey), **Ir** + TEOA (pink) , and **Ir** + BrAPN (blue) in acetonitrile after excitation at 470 nm; a 550 nm long pass filter was used for detection. Concentrations of species were as follows: [Ir] = 31.2 µM, [BrAPN] = 12 mM, and [TEOA] = 36 mM. Data was fit to a single function (solid lines); details can be found in Table S5.

Table S5. TCSPC fits for heterogenized catalysts, homogeneous **Ir**, and **Ir** in the presence of TEOA and BrAPN. ITO-**Ir** and Al_2O_3-**Ir** were fit to a biexponential function, while the others were fit to a single exponential function. All fits confine y_0 to 0.

**Sample**	**A_1**	**τ_1 (ns)**	**A_2**	**τ_2 (ns)**	**$\tau_{average}$ (ns)**[c]
ITO-**Ir**[a]	770	1.8	99	130	1.5
Al_2O_3-**Ir**[a]	410	1.7	410	150	3.3
ZrO_2-**Ir**	900	220	-	-	-
**Ir**[b]	880	210	-	-	-
**Ir**+TEOA[b]	890	80	-	-	-
**Ir**+BrAPN[b]	890	180	-	-	-

[a]samples did not provide good fits to a single exponential function, and were thus, fit to biexponential functions; surface inhomogeneity or non-innocent surface behavior could be reasons for the need to add an extra component to the fits. [b]solution phase samples dissolved in acetonitrile. [c] calculated using the weighted average of logarithmic lifetimes (ref.[8])

Photoluminescence Measurements

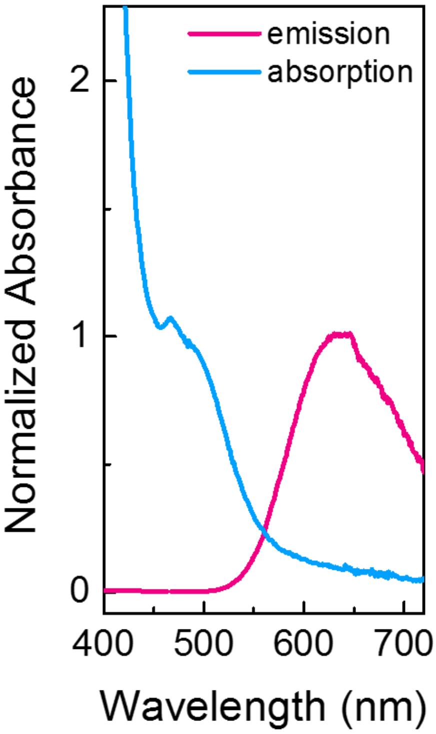

Figure S36. UV-Vis absorption (blue) and photoluminescence (pink) spectra for **Ir** in acetonitrile. **Ir** was excited at 370 nm to collect the photoluminescence spectrum. The spectra were normalized to the bands at 635 nm (emission) and 497 nm (absorption). The spectra normalized at the maximum of the lowest energy absorption band, were found to intersect at 559 nm (2.21 eV), which was used to estimate the excited state potentials of **Ir** using Weller approximations.[9]

References:

[1] a) K. S. Kjaer, N. Kaul, O. Prakash, P. Chabera, N. W. Rosemann, A. Honarfar, O. Gordivska, L. A. Fredin, K. E. Bergquist, L. Haggstrom, T. Ericsson, L. Lindh, A. Yartsev, S. Styring, P. Huang, J. Uhlig, J. Bendix, D. Strand, V. Sundstrom, P. Persson, R. Lomoth, K. Warnmark, *Science* **2019**, *363*, 249-253; b) A. El-Zohry, A. Orthaber, B. Zietz, *J. Phys. Chem. C Nanomater. Interfaces* **2012**, *116*, 26144-26153.

[2] J. B. Waern, C. Desmarets, L. M. Chamoreau, H. Amouri, A. Barbieri, C. Sabatini, B. Ventura, F. Barigelletti, *Inorg. Chem.* **2008**, *47*, 3340-3348.

[3] K. L. Materna, B. Rudshteyn, B. J. Brennan, M. H. Kane, A. J. Bloomfield, D. L. Huang, D. Y. Shopov, V. S. Batista, R. H. Crabtree, G. W. Brudvig, *ACS Catal.* **2016**, *6*, 5371-5377.

[4] S. A. Trammell, T. J. Meyer, *J. Phys. Chem. B* **1999**, *103*, 104-107.

[5] W. G. McGimpsey, J. C. Scaiano, *Canadian Journal of Chemistry* **1988**, *66*, 1474-1478.

[6] H. Mo, D. Raftery, *Anal. Chem.* **2008**, *80*, 9835-9839.

[7] K. L. Materna, R. H. Crabtree, G. W. Brudvig, *Chem. Soc. Rev.* **2017**, *46*, 6099-6110.

[8] L. Zhang, L. Favereau, Y. Farre, E. Mijangos, Y. Pellegrin, E. Blart, F. Odobel, L. Hammarstrom, *Phys Chem Chem Phys* **2016**, *18*, 18515-18527.

[9] A. Weller, *Zeitschrift für Physikalische Chemie* **1982**, *133*, 93-98.
